# Enhancing EEG-Based Brain Pattern Recognition Through Functional-Network-Level Volume Conduction Mitigation: Spatially Informed Decay Modeling–Residual Correction

**DOI:** 10.3390/brainsci16060649

**Published:** 2026-06-18

**Authors:** Yuzeng Xu, Sho Otsuka, Seiji Nakagawa

**Affiliations:** 1 Graduate School of Science and Engineering, Chiba University, 1-33, Yayoi-cho, Inage-ku, Chiba 263-8522, Japan; yuzeng-xu@chiba-u.jp; 2 Center for Frontier Medical Engineering, Chiba University, 1-33, Yayoi-cho, Inage-ku, Chiba 263-8522, Japan; otsuka.s@chiba-u.jp; 3 Graduate School of Engineering, Chiba University, 1-33, Yayoi-cho, Inage-ku, Chiba 263-8522, Japan; 4 Department of Medical Engineering, Faculty of Engineering, Chiba University, 1-33, Yayoi-cho, Inage-ku, Chiba 263-8522, Japan; 5 Med-Tech Link Center, Chiba University Hospital, 1-8-1, Inohana, Chuo-ku, Chiba 260-8677, Japan

**Keywords:** EEG, brain–computer interface, functional connectivity, affective computing, deep learning

## Abstract

**Highlights:**

**What are the main findings?**

- A spatially informed decay modeling–residual correction (DM-RC) method was proposed to mitigate volume conduction effects directly at the EEG functional network level.
- The VC-mitigated functional networks improved brain pattern recognition performance compared with the observed functional networks, especially when evaluated through subnetwork-based classification.

**What are the implications of the main findings?**

- The findings suggest that distance-dependent volume conduction components can be explicitly modeled and suppressed without reconstructing cortical sources or reprocessing raw EEG signals.
- DM-RC provides a computationally feasible post hoc refinement strategy for functional-connectivity-based brain–computer interface and brain pattern recognition pipelines.

**Abstract:**

**Background/Objectives:** Advancements in neuroscience and machine learning have increasingly enabled brain pattern recognition based on bio-signal measurements, such as electroencephalography (EEG). These developments support next-generation technologies, including brain–computer interfaces (BCIs) and AI-assisted systems. However, volume conduction (VC) effects remain a major source of contamination in EEG recordings, affecting both univariate analyses and functional connectivity estimation. **Methods:** In this work, we propose a VC mitigation method that explicitly models and suppresses VC components in the observed functional networks. Specifically, the observed functional network is decomposed into a matrix capturing only VC-related components (i.e., components attributed to volume conduction) and a residual matrix, where the residual is regarded as a proxy for a VC-mitigated functional network that better reflects the underlying functional interactions. The VC component matrix is modeled using a decay function parameterized by the inter-electrode distance matrix, capturing the dominant spatial bias induced by VC. To estimate these parameters, we introduce supervised channel importance, quantified as the mutual information between experimental labels and channel signals, as a proxy for task-relevant neural activity. The parameters are optimized such that the unsupervised node importance derived from the VC-mitigated functional network, defined as the average node strength, aligns with the supervised channel importance. **Results:** Evaluation results using a deep-learning framework demonstrate that, compared with the observed functional network, the VC-mitigated functional network improves classification performance in brain pattern recognition tasks.

## 1. Introduction

Advancements in neuroscience and machine learning have accelerated the development of automated brain pattern recognition. Typically implemented through task classification, this process relies on bio-signals such as electroencephalography (EEG), magnetoencephalography, and eye tracking [1,2,3,4,5]. These breakthroughs are foundational for next-generation technologies, including brain–computer interfaces (BCIs) [6] and AI-assisted systems [7].

Functional connectivity, which quantifies the statistical dependence between neural signals across brain regions, has emerged as a core paradigm for brain pattern analysis [8,9]. The derived functional networks are often represented as adjacency matrices and used as input samples for pattern recognition models, such as convolutional neural networks (CNNs), for cognition decoding [10].

Former studies and applications have increasingly paired non-invasive modalities like EEG with functional connectivity analysis [2,9,10,11]. This approach offers both practical feasibilities, due to the cost-effectiveness of EEG, and rich spatiotemporal data for high-performance BCIs.

However, volume conduction (VC) effects on functional connectivity pose a critical challenge [12,13]. VC refers to the passive spread of electromagnetic fields through biological tissue, resulting in signal mixing across sensors that may not reflect neuronal interactions [13]. These effects can induce spurious correlations and phase synchrony between electrodes in functional connectivity estimation [8]. Such contamination is especially problematic when the functional networks are used as input samples for machine learning, as they can obscure the underlying neurophysiological structure and further degrade recognition performance.

This calls for VC mitigation methods in functional connectivity analysis. Here, we propose a low-cost, spatially modeled VC mitigation method that explicitly models and suppresses VC components in the originally observed functional networks. Specifically, the observed functional network is decomposed into a matrix only capturing VC components and a residual matrix, where the residual is interpreted as the VC-mitigated functional network that more faithfully reflects underlying functional interactions.

**Figure 1:**
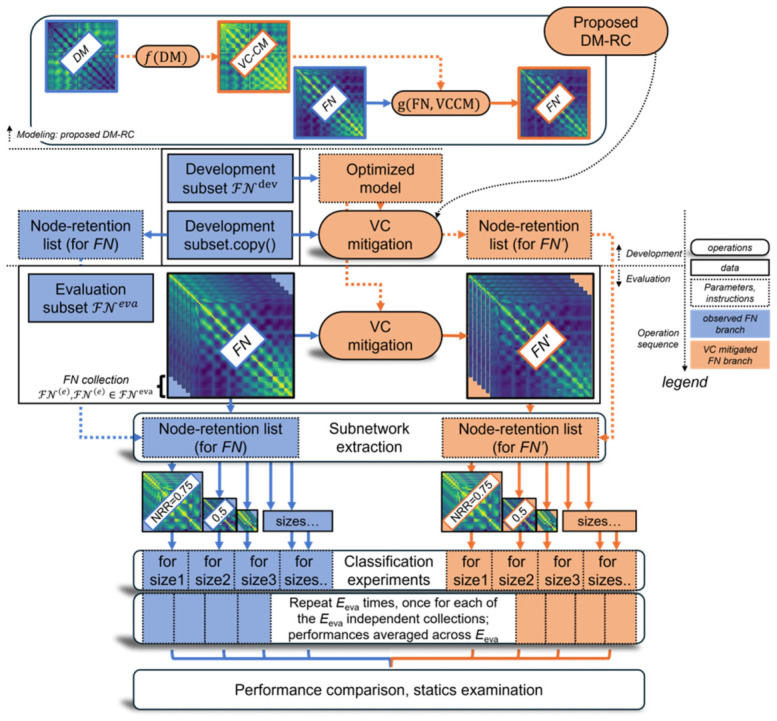
Illustration of the overall framework of this study, highlighting modeling, dataset partitioning, and the evaluation framework. Legend: *FN:* observed functional network; *FN’:* VC-mitigated functional network; DM-RC: the proposed method of decay modeling–residual correction; *DM:* inter-electrode distance matrix; *VC-CM:* VC component matrix; *f*(⋅) and *g*(⋅): functions. The upper branch represents the development subset, which was jointly used for model optimization and construction of the node-retention list for subnetwork extraction. The lower branch depicts the evaluation subset, which was reserved exclusively for classification experiments. This design enforces strict isolation between the two branches and effectively prevents data leakage. Performance was compared across different methods and varying subnetwork sizes.

The effectiveness of the proposed method was evaluated by examining whether mitigation improves classification performance in actual brain pattern recognition, thereby indicating the enhanced discrimination of functional networks. Subnetworks were extracted through node retention as the input samples, and classification performance was assessed using convolution neural networks (CNNs). The results show that the VC-mitigated networks significantly improve classification accuracy, particularly under sparse node retention conditions. Th overall framework is illustrated in Figure 1.

## 2. Related Works on VC Mitigation

### 2.1. Blind Source Separation and Source Reconstruction

Blind source separation VC mitigation methods [14], most notably ICA, attempt to isolate statistically independent sources, thereby reducing signal mixing [15]. ICA is widely used to remove global artifacts (e.g., eye blinks), but its effectiveness in resolving overlapping neuronal sources is limited by the assumption of statistical independence and the lack of anatomical specificity. When not combined with source localization, ICA-derived components can also be difficult to interpret in terms of brain regions.

Source reconstruction methods (e.g., LORETA, and beamformers [16,17]) project sensor-level signals into cortical source space using MRI-informed forward models. While these methods enhance anatomical interpretability and partially reduce VC, they are highly sensitive to model inaccuracies and require substantial computational and anatomical resources, such as individual MRI scans and high-density electrode arrays.

### 2.2. Sensor-Level Methods

Sensor-level methods, such as SLF [18], are designed to enhance local signal components while suppressing spatially diffuse activity. Although conceptually straightforward, these methods must be applied prior to functional connectivity estimation, which imposes several practical limitations.

First, operating to the raw signals makes these methods incompatible with pipelines in which functional connectivity has already been computed or embedded, such as pretrained models, offline feature banks, or standardized benchmark datasets. In such cases, sensor-level processing cannot be retroactively integrated.

Second, sensor-level methods intrinsically alter the raw signal representation through spatial projection or suppression, which may lead to irreversible information loss [19]. This transformation constrains the flexibility of subsequent feature engineering stages, including feature enhancement and feature fusion. When such downstream operations are required, reprocessing from the raw signal may become necessary, introducing redundant computation and additional pipeline complexity.

Third, many sensor-level methods rely on dense and consistent electrode configurations to ensure reliable spatial filtering. This assumption may not hold in large-scale or heterogeneous datasets with varying montages, missing channels, or nonuniform sensor layouts, thereby limiting their scalability and practical applicability.

### 2.3. Network-Level Methods

Another family of methods addresses VC at the functional network level.

A group of straightforward methods relies on phase-based connectivity metrics, such as imaginary coherence [20], the phase lag index (PLI) [21], and its variants (e.g., weighted and debiased PLI, and temporal irreversibility index [22]). These methods discard or downweight zero-lag coupling, which is typically associated with VC. While effective at reducing spurious synchrony, they also ignore underlying zero-lag interactions and are often sensitive to noise and limited data length, especially in fast-paced or short-window computation scenarios.

In parallel, although graph signal processing (GSP) has not been originally developed to explicitly model and then analyze VC, its mathematical principles are inherently well-aligned with functional network analysis [23]. Classical GSP methods, particularly graph Laplacian filtering (GLF) [24] in a high-pass formulation that suppresses smooth or low-spectral-frequency components on the graph, should be reconsidered as effective candidates for enhancing functional connectivity. Such operations act directly on the connectivity matrix and promote locally specific interactions while attenuating spatially diffuse coupling, which is a pattern that largely overlaps with typical VC components.

Recent studies have applied graph signal analysis [22,25], graph learning [26,27,28], and related GSP frameworks (e.g., graph theory and hidden Markov modeling [29]) to characterize functional connectivity and neurological disorders, further supporting the suitability of graph-domain processing for EEG functional network analysis, both of them on the sensor level or network level. Therefore, integrating GSP-based high-pass methods provides a theoretically grounded yet practically flexible avenue for the post hoc refinement of functional networks without modifying the raw signals.

### 2.4. Reasonable Pipeline of Comparison

While various VC mitigation strategies have been proposed, they differ substantially in their assumptions and practical requirements, with inherent limitations. These limitations become more critical in BCI applications, where real-time operation imposes strict constraints on computational cost and temporal window length. Methods requiring large numbers of temporal samples, extensive time-domain processing, complex optimization, or forward modeling are often difficult to integrate into practical BCI pipelines. Thus, although existing approaches can partially mitigate VC, none fully resolves it, especially under real-time BCI conditions.

Given these constraints, this study focuses on methods that are more feasible for functional-connectivity-based BCI pipelines. Among existing approaches, SLF and SSD are included as sensor-level competitors because they require relatively few additional anatomical or computational resources. Since the proposed method operates directly on functional networks rather than raw signals, network-level approaches provide the most relevant benchmarks. Accordingly, we include graph Laplacian filtering (GLF) as a network-level competitor and further extend SLF to the network domain, resulting in generalized SLF (GSLF).

To ensure a fair comparison, this study evaluates three categories of functional networks: (1) the originally observed functional networks as the baseline, (2) VC-mitigated functional networks obtained using competing methods, including SLF, SSD, GLF, and GSLF, and (3) functional networks optimized using the proposed method.

## 3. Competing Methods

### 3.1. Surface Laplacian Filtering

SLF is a sensor-level method commonly used to reduce VC effects in EEG by enhancing spatially local neural activity and attenuating broadly distributed components [18]. It improves the spatial resolution and supports more reliable functional connectivity estimation.

SLF can be expressed as a weighted local subtraction:

(1) $$V^{\prime }\left(i\right)=V\left(i\right)-\sum_{k\in N\left(i\right)}W_{i,k}V\left(k\right),$$

where *V*(*i*) denotes the potential at electrode *i*, *N*(*i*) is the neighborhood of electrode *i*, and *W_i,k_* are spatial weights determined from the inter-electrode distance matrix *DM* using a Gaussian kernel:

(2) $$W_{i,k}=\exp\left(-\frac{{DM}_{i,k}^{2}}{2\sigma^{2}}\right),W_{i,i}=0.$$

The spatial weights excluding self-connections are row-normalized as follows:

(3) $$W_{i,j}\leftarrow \frac{W_{i,j}}{\sum_{j}W_{i,j}}.$$

In our practice, SLF was implemented using a spherical spline interpolation, which computes the spatial Laplacian directly from electrode coordinates on a unit sphere without explicit neighborhood construction. The spline order was set to *m* = 4, and a Tikhonov regularization term *λ* = 10^−4^ was applied. The spherical harmonic expansion order was automatically determined based on the number of electrodes.

SLF was applied once to the broadband EEG signals, after which band-specific signals were obtained via standard band-pass filtering. All subsequent analyses including connectivity estimation were performed on the filtered data.

### 3.2. Spatial Spectral Decomposition

SSD is a sensor-level method that enhances activity in a target frequency band by maximizing its variance relative to neighboring frequency bands [30,31]. It is formulated as a generalized eigenvalue problem on band-limited covariance matrices.

Given multichannel signals $X\in R^{T\times C}$ where *T* and *C* denote the number of temporal samples and channels, respectively, the signal covariance *Cs* is computed from data filtered in the target band [*f*1, *f*2], and the noise covariance *Cn* is defined as the average covariance over *K* flanking bands:

(4) $$C_{s}=cov\left(X_{\left[f_{1},f_{2}\right]}\right),C_{n}=\frac{1}{K}\sum_{k=1}^{K}cov\left(X_{\mathrm{noise},k}\right).$$

Here, *X_noise,k_* is the data filtered in the *k*-th band outside [*f*1, *f*2], typically placed symmetrically to capture broadband background activity; *K* is the number of such bands.

To ensure well-conditioned covariances, shrinkage regularization is applied:

(5) $$C_{s}\leftarrow C_{s}+\epsilon \frac{tr(C_{s})}{C_{s}}I,{\;C}_{n}\leftarrow C_{n}+\epsilon \frac{tr(C_{n})}{C_{n}}I,$$

where *ϵ* > 0 is a small constant, *tr*(⋅) is the trace, and $I\in R^{C\times C}$ is the identity matrix.

The SSD filters *w* are obtained from the following:

(6) $$C_{s}w=\lambda C_{n}w.$$

Eigenvectors with the largest eigenvalues form *W*, yielding components *S = W*^⊤^ *X*. Spatial patterns are *A* = (*W*^†^)^⊤^, and reconstructed signals:

(7) $$X^{\prime }=A\cdot S=(W^{†}{)}^{⊤}\cdot W^{⊤}X.$$

In our practice, zero-phase Butterworth filters define signal/noise bands, regularized covariances (*ϵ* = 10^−3^) and whitening-based eigen-decomposition are used, and six components are retained per band. In this pipeline, SSD replaces conventional band-pass filtering.

### 3.3. Generalized Surface Laplacian Filtering

GSLF, as we defined here, extends the surface Laplacian principle from signals to functional connectivity networks by applying a Laplacian-like operation in the edge domain.

Given a functional network *FN*, where *FN*(*i,j*) denotes the connectivity between electrodes *i* and *j*, the GSLF and the VC-mitigated network *FN’* are defined as follows:

(8) $${FN}^{\prime }\left(i,j\right)=FN\left(i,j\right)-\sum_{\left(k,l)\in N(i,j\right)}W_{\left(i,j\right),\left(k,l\right)}FN\left(k,l\right),$$

where *N*(*i*,*j*) denotes the edge-domain neighborhood consisting of edge pairs that share at least one endpoint with (*i*,*j*).

Edge-domain weights are constructed by extending the inter-electrode distances:

(9) $$W_{\left(i,j),(k,l\right)}=\exp\left(-\frac{\left(DM_{i,k}+DM_{j,l}{)}^{2}\right.}{2\sigma^{2}}\right),W_{\left(i,j),(i,j\right)}=0,$$

and followed by row-normalization to ensure the unit sum across neighboring edges. The resulting operation suppresses smoothly distributed connectivity components while preserving the localized edge-level structure.

### 3.4. Graph Laplacian Filtering

GLF applies Laplacian-based filtering directly to functional networks by modeling them as weighted graphs [23,24].

The graph adjacency matrix is defined using a Gaussian kernel on the inter-electrode distance matrix:

(10) $$W_{i,j}=exp(-\frac{{DM}_{i,j}^{2}}{2\sigma^{2}}),W_{i,i}=0,$$

and followed by row-normalization. The degree matrix *D* and normalized graph Laplacian are given by the following:

(11) $$D_{i,i}=\sum_{j}W_{i,j},L=I-D^{-\frac{1}{2}}WD^{-\frac{1}{2}}.$$

First-order graph filters are defined as follows:

(12) $$F=\left\{\begin{array}{cc} I-\alpha L, & \mathrm{low-pass},\\ \alpha L, & \mathrm{high-pass}, \end{array}\right.$$

where ***α*** controls the filtering strength. The unilateral GLF and the filtered functional network, considered the VC-mitigated functional network, are obtained as follows:

(13) $$FN^{\prime }=F\cdot FN.$$

## 4. Proposed Method

The structural similarity between EEG connectivity matrices and the inter-electrode distance matrix motivates the central hypothesis of this study: a substantial portion of this similarity is driven by the VC effects.

Based on this insight, we propose a computationally efficient VC mitigation method named decay modeling–residual correction (DM-RC) that explicitly models the VC component as a function of the inter-electrode distance and suppresses the components directly at the functional network level. By parameterizing VC effects rather than reprocessing the raw time-series signals, the method enables the flexible post hoc refinement of the precomputed functional connectivity and facilitates the seamless integration into practical BCIs.

### 4.1. Decay Modeling and VC Components

VC represents the passive propagation of electrical fields through biological tissues. In non-invasive recordings such as EEG, this effect causes sensor signals to reflect mixtures of nearby neuronal activities rather than spatially isolated generators, owing to the relatively high conductivity of the brain tissue [8,12,13].

Although the precise characterization of intracranial transmission typically requires complex brain models, the present study adopts a practical simplification for BCIs, aiming to capture the dominant contamination with minimal engineering demands. The VC component between any pair of electrodes is modeled as a function *f*(⋅) of their distance *d*, and is normalized to the range [0, 1] within the distance matrix:

(14) VC=f(d),

In this study, distances were calculated using the Euclidean distance between EEG electrode locations. Although geodesic distances along the scalp surface may more accurately reflect the signal propagation on the scalp, the Euclidean distance was adopted because volume conduction primarily occurs through intracranial tissues rather than exclusively along the scalp surface.

To characterize the decay of the VC strength, we employed several decay functions, including exponential, rational quadratic, generalized Gaussian, and sigmoid. These functions are summarized in Table 1.

For a given electrode configuration, the VC component between any two electrodes *ei* and *ej* is modeled as follows:

(15) $$\mathrm{VC}(e_{i},e_{j})=f(d(e_{i},e_{j})),$$

This formulation induces the components into a VC component matrix (VC-CM):

(16) $${V\mathrm{C-CM}}_{i,j}=f\left({\mathrm{DM}}_{i,j}\right),$$

where *i, j* ∈ {1, 2, …, *C*} index the EEG channels/electrodes, $\mathrm{DM}\in R^{C\times C}$ denotes the inter-electrode distance matrix whose entries represent the spatial distances between electrode pairs, and $\mathrm{VC-CM}\in R^{C\times C}$ corresponding to $\mathrm{DM}\in R^{C\times C}$.

### 4.2. Residual Correction

The corrected functional network from VC (or the VC-mitigated functional network) is defined as the residual between the observed functional network and estimated *VC-CM*:

(17) $$FN^{\prime }=FN-k\;V\mathrm{C-CM}=g\left(FN,V\mathrm{C-CM}\right),$$

where $\mathrm{FN}\in R^{C\times C}$ denotes the observed functional network, ${\mathrm{FN}}^{\prime }\in R^{C\times C}$ denotes the VC-mitigated functional network, and *k* is a global scaling parameter that controls both the magnitude and sign of the artifact contribution.

Since the *VC-CM* is generated from the *DM* through the decay function defined in (16), the correction can be expressed as follows:

(18) $$FN^{\prime }=g\left(FN,f\left(DM\right)\right)=g\left(FN,f\left(DM;\Theta_{f}\right);\Theta_{g}\right)=M(FN,DM;\Theta ),$$

where *g*(⋅) represents the correction operation of (17), and M(⋅) denotes the overall modeling process from *FN* and *DM* to the *FN’*, using the parameter set *Θ*.

**Figure 2:**
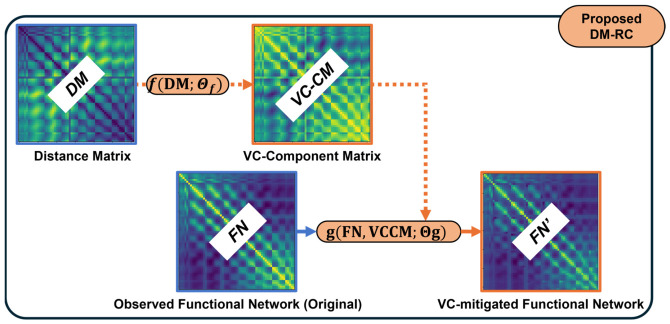
Illustration of the proposed method of DM-RC. This method takes the inter-electrode distance matrix *DM*, the observed functional network *FN*, a decay function *f*(⋅), a correction operation *g*(⋅), and parameters *Θ* as inputs, and outputs the VC-mitigated functional network *FN’*. The spatially smooth VC components *VC-CM* is first modeled, and the VC-mitigated functional network is then obtained by subtracting the scaled *VC-CM* from the observed functional network, resulting in a residual matrix that more closely reflects underlying functional interactions.

The set *Θ =* {*σ*, *α*, *β*, *μ*, *k*} contains the model parameters for the decay function described in (14) and listed in Table 1, as well as the parameters *k* for the correction operation in (17). Specifically, *Θ_f_*, *Θ_g_* ⊆ *Θ*, where *Θ_f_* = {*σ*, *α*, *β*, *μ*} and *Θ_g_* = {*k*}, denote the parameter sets for the decay function *f*(⋅) and the correction operation *g*(⋅), respectively, as shown in (18). This integrated modeling process is illustrated in Figure 2.

### 4.3. Model Assumptions and Limitations

It should be noted that the proposed distance-based modeling represents a simplified approximation of VC rather than a physiologically complete description of intracranial electrical propagation. The model assumes that the inter-electrode distance is the dominant factor governing the spatial distribution of VC contamination, while other factors, such as tissue conductivity heterogeneity, head geometry, source orientation, and individual anatomical variability, are not explicitly considered. Consequently, the estimated VC component should be interpreted as an engineering approximation designed to capture the major distance-dependent VC characteristics, rather than a direct reconstruction of the underlying biophysical process.

## 5. Measurements for Functional Connectivity

Functional networks of the brain can be constructed using various connectivity measurements, such as the Pearson correlation coefficient (PCC), phase-locking value (PLV), phase-locking index, mutual information, and others [8]. In this study, PCC and PLV were employed as measures of functional connectivity due to their widespread use and computational simplicity.

For time-series data such as EEG signals *xi* and *xj* from different EEG channels *i* and *j*, the PCC quantifies the linear relationship between the two signals and is defined as follows:

(19) $$PCC\left(x_{i},x_{j}\right)=\frac{\sum_{t=1}^{T}\left(x_{i,t}-\bar{x_{i}}\right)\left(x_{j,t}-\bar{x_{j}}\right)}{\sqrt{\sum_{t=1}^{T}{\left(x_{i,t}-\bar{x_{i}}\right)}^{2}}\sqrt{\sum_{t=1}^{T}{\left(x_{j,t}-\bar{x_{j}}\right)}^{2}}},$$

where $\bar{x_{i}},\bar{x_{j}}$ are the respective means, *t* indexes the time samples, and *T* denotes the total number of samples.

The PLV measures the phase synchronization strength between two signals across time:

(20) $$PLV(x_{i},x_{j})=|\frac{1}{T}\sum_{t=1}^{T}e^{j(\phi_{x_{i}}(t)-\phi_{x_{j}}(t))}|,$$

where $\phi_{x_{i}}\left(t\right)$ and $\phi_{x_{j}}\left(t\right)$ are the instantaneous phases of the two signals, $j=\sqrt{-1}$.

The PCC value ranges from −1 (perfect negative correlation), to 0 (no linear relationship), to 1 (perfect positive correlation). The PLV value ranges from 0 (no synchronization) to 1 (perfect phase locking).

## 6. Dataset and Feature Engineering

### 6.1. EEG Dataset

The method evaluation for brain pattern recognition was conducted using the SJTU Emotion EEG Dataset (SEED) [32], a widely adopted benchmark for emotion recognition studies. The dataset comprises 62-channel 200 Hz EEG experimental recordings collected according to the international 10–20 system from fifteen subjects, each of whom participated in three independent experiments, resulting in 45 EEG recordings of the same length in total.

In each experiment, subjects viewed fifteen pre-labeled video clips (approximately four minutes per clip, total 3394 s) designed to elicit emotions across three valence categories: negative, neutral, and positive. Each recording was annotated with the corresponding valence labels.

Consequently, the dataset used in this study consists of 45 EEG recordings, each with 62 channels, a sampling rate of 200 Hz, and a duration of 3394 s, together with the corresponding emotional valence annotations.

### 6.2. EEG Preprocessing

The preprocessing followed the SEED developers: the raw data were downsampled to a sampling rate of 200 Hz, and the signals were visually inspected. Segments contaminated by electromyography (EMG) and electrooculography (EOG) artifacts were manually removed. The data were then processed using a band-pass filter from 0.3 Hz to 50 Hz to reduce noise and artifacts.

The preprocessed EEG signals were filtered into five classical frequency bands: delta (1–4 Hz), theta (4–8 Hz), alpha (8–13 Hz), beta (13–30 Hz), and gamma (30–45 Hz). Among these, the alpha, beta, and gamma bands were selected for feature extraction, as higher-frequency activity is more strongly associated with emotional processing [32].

### 6.3. EEG Representation

Formally, the EEG dataset consisting of *E* recordings can be represented as

(21) $$EEG\in R^{E{\times (Lf}_{s})\times C}$$

where *C* is the number of channels, *L* is the duration of each recording in seconds, and *fs* is the sampling rate in Hz. The product *Lfs* represents the total number of time samples per recording; this is treated as a single unified temporal dimension rather than two separate parameters, maintaining structural parity with the recording *E* and channel *C* dimensions.

Specifically, the multichannel EEG signal and the single-channel EEG signal from the ***e***-th recording are defined as

(22) $$x^{\left(e\right)}={EEG}^{\left(e\right)}=EEG\left[e,:,:\right],x^{\left(e\right)}\in R^{{(Lf}_{s})\times C},$$

(23) $${x_{i}^{\left(e\right)}=EEG}^{\left(e\right)}\left[e,:,i\right],x_{i}^{\left(e\right)}\in R^{{Lf}_{s}},$$

where *e* ∈ {*1*, *2*, …, *E*} indexes the recordings in the EEG dataset, and *i* ∈ {*1*, *2*, …, *C*} index the channels.

The signals were then segmented into 1 s non-overlapping windows, yielding *N* windows with *T* data points in each window. Thus, the recording duration *L* in seconds is equal to the number of windows; the number of data points *T* is equal to *fs*—i.e., *N* = *L*; *fs* = *T*. The segmented EEG dataset can be described as follows:

(24) $$EEG\in R^{E\times (NT)\times C}$$

For windows *n* ∈ {1, 2, …, N}, the signal in the *n*-th window is defined as follows:

(25) $$x_{n,i}^{\left(e\right)}=EEG\left[e:\left(n-1\right)T:nT,i\right],x_{n,i}^{\left(e\right)}\in R^{T},$$

where $x_{n,i}^{\left(e\right)}$ denotes the signal from channel *i* in the *n*-th window of recording *e*. This windowed signal $x_{n,i}^{\left(e\right)}$ is used to estimate the functional connectivity shown in (19) and (20).

### 6.4. Representation of Functional Networks

The correspondence between EEG and functional networks is shown in the column “Dataset” in Table 2.

Functional connectivity was estimated independently within each subject, recording, frequency band, and temporal window. During this estimation step, no cross-subject, cross-recording, cross-band, or cross-window pooling, averaging, or aggregation was performed.

Each recording was segmented into *N* windows. Within each window, functional connectivity was extracted across the *C* EEG channels to construct a functional brain network. This operation was performed separately for each of the *B* frequency bands, where *B* = 3 corresponds to the alpha, beta, and gamma bands.

Consequently, the collection of functional networks (or the tensor of functional connectivity matrices) ${FN}^{\left(e\right)}$ corresponding to the *e*-th recording *x^(e)^* (22) is defined as follows:

(26) $${FN}^{\left(e\right)}\in R^{N\times B\times C\times C}.$$

In this representation of (26), *N* denotes the number of functional network samples, B refers to frequency bands, and *C × C* represents the dimension of single functional network map (connectivity matrix).

Each recording *x^(e)^* corresponds to one collection of functional networks ${FN}^{\left(e\right)}$. Thus, for the entire dataset, the dimensionality of the functional network collections is

(27) $$FN\in R^{E\times N\times B\times C\times C},$$

where *E* refers to the number of collections, corresponding to the number of EEG recordings (21) and (24).

### 6.5. Interface to Classification Experiments

The correspondence between tensors for classification experiments and functional networks is shown in the row “Deep learning framework for classification experiments” in Table 2.

From a machine-learning/deep-learning perspective, the representation in (26) can be regarded as a four-dimensional tensor. Specifically, the dataset (27) contains *E* such tensors, where each tensor corresponds to an independent classification experiment. For each tensor, *N* denotes the number of samples, *B* denotes the number of feature channels, and *C* × *C* denotes the size of each feature map for a single sample.

A minimal functional network map (or a connectivity matrix) is defined as follows:

(28) $${{FN}^{\left(e,b,n\right)}\in R^{C\times C},{FN}^{\left(e,b,n\right)}=FN}^{\left(e\right)}[n,b,:,:],$$

which represents the functional network estimated from the *n*-th window and the *b*-th frequency band of the *e*-th recording.

The *n*-th sample used as input to the deep-learning model is composed of *B* frequency-specific functional connectivity maps:

(29) $${{FN}^{\left(e,n\right)}\in R^{B\times C\times C},{FN}^{\left(e,n\right)}=FN}^{\left(e\right)}[n,:,:,:].$$

### 6.6. Data Partitioning

The data partitioning strategy is summarized in Table 2 and illustrated in Figure 1.

To prevent data leakage, the dataset *EEG*, consisting of recordings from fifteen subjects with three independent experiments each, was partitioned into two subsets. Data from five subjects (15 recordings) were used as a single development subset *EEG*_dev_ for both model optimization and the subsequent construction of the node-retention list employed in the subnetwork extraction of functional networks, without further subdivision. The remaining data from ten subjects (30 recordings) were reserved as evaluation subset *EEG*_eva_ for downstream classification experiments. The corresponding collections of functional networks are ${FN}^{dev}$ and ${FN}^{eva}$.

The 5:10 partition was adopted to balance parameter optimization and evaluation reliability. Because the proposed models contain only a limited number of parameters and the node-retention list is derived from average network characteristics, a small development subset is sufficient, while reserving more subjects for evaluation improves the robustness and generalizability of the results.

Each collection ${FN}^{\left(e\right)}$ in the evaluation subset ${FN}^{eva}$ serves as the sole input to one classification experiment, resulting in 30 independent collections.

## 7. Evaluation Framework

Emotion recognition is a core topic in BCI research, aiming to infer an individual’s emotional state from biological signals such as EEG. Its task-driven paradigm is well-suited to the requirements of the evaluation framework in this study: active neural states yield a sufficiently strong VC effect for operational modeling and discrimination; and the availability of explicit labels provides clear quantitative indices for performance evaluation; and emotion recognition is known to rely heavily on functional connectivity patterns rather than on isolated neural activities, making it an appropriate and sensitive application scenario for assessing the quality of the recovered dynamic functional networks.

The effectiveness of the proposed method was evaluated by examining whether mitigation improves classification performance in actual brain pattern recognition, thereby indicating the enhanced discrimination of functional networks. Subnetworks were extracted through node retention as the input samples, and classification performance was assessed using CNNs. This framework is illustrated in Figure 1.

### 7.1. Subnetwork Extraction

Comparisons based only on full-size functional networks may not fully reveal performance differences between methods, since redundant or weakly informative connections can obscure differences in discriminative information density. To provide a more refined comparison across different network scales, a subnetwork extraction scheme was introduced.

After the construction of functional networks in (26) and (27), subnetworks of different sizes were extracted and used as input samples for the classification experiments. Subnetwork extraction was guided by a node-retention list constructed from node importance scores. Specifically, nodes were ranked according to their importance, and the top-ranked nodes determined by the node-retention rate (NRR) were retained. The size of a single subnetwork is represented as (*NRR* × *C*) × (*NRR* × *C*).

The calculation of node importance *NI* has been defined in (30) and (31).

(30) $$NI={mean}_{col}\left(FN\right),NI\in R^{C},$$

(31) $${NI}^{{FN}^{\prime }(\Theta )}={mean}_{col}\left({FN}^{\prime }(\Theta )\right),{NI}^{{FN}^{\prime }(\Theta )}\in R^{C}.$$

Node retention lists were constructed exclusively on the development subset, which was strictly isolated from subsequent classification experiments. Although both parameter optimization and node-retention list construction were performed on the same subset without further subdivision, the node-retention lists were individually constructed for each method after individual parameter optimization and VC mitigation using optimal parameters. The node importance for a given method is defined as the following:

(32) $${NI}^{{\langle FN\rangle }^{\prime },method}={mean}_{col}\left({\langle {FN}^{\prime }\rangle }^{\left(method\right)}\right).$$

Node-retention lists differed across methods because node importance was computed independently for each connectivity representation. This design allowed each method to be evaluated using the network topology it produced, while maintaining a consistent extraction criterion within each branch and avoiding the imposition of a unified node list that would be incongruent with the processed connectivity matrices.

### 7.2. Balanced-Performance Efficiency

Reporting accuracy at only a single subnetwork ratio can be misleading, as a method may perform well at that ratio but degrade substantially for smaller subnetworks, which are precisely the configurations that yield computational savings.

Therefore, the area under the curve (AUC) was employed, which comprehensively evaluates dependent-variable values across the range of the independent variable; here, these correspond to classification accuracy and subnetwork size (NRR), respectively.

Building upon the classical AUC metric, balanced-performance efficiency (BPE) is proposed as a comprehensive measure that captures not only the overall classification accuracy, as reflected by the AUC, but also the computational efficiency at reduced subnetwork sizes. To achieve this, a reward is assigned to performance that exceeds a linear performance-degradation expectation:

(33) $$R\left(k\right)=\rho \left(k\right)\cdot \left(Acc\left(k\right)-Acc\left(k_{0}\right)\cdot k\right)={(e}^{-k}-e^{-1})\cdot (Acc\left(k\right)-Acc(k0)\cdot k),$$

where *Acc*(*k*_0_)*·k* represents the linear expectation of accuracy under network sparsification. This expectation assumes that performance decreases proportionally with the reduction in subnetwork size. Any performance exceeding this linear expectation is rewarded. The reward coefficient *ρ*(*k*) is defined as follows:

(34) $$\rho \left(k\right)=e^{-k}-e^{-1}$$

where the term *e^−k^* introduces a decreasing reward as the NRR increases, thereby emphasizing the performance at smaller subnetworks. The subtraction of *e*^−1^ ensures that the reward coefficient becomes zero when the full network is used (*k* = 1), providing a balanced reference point.

At a given NRR *k*, the single-point balanced-performance efficiency (SBPE) is then defined by giving the reward to accuracy:

(35) $$SBPE\left(k\right)=R\left(k\right)+Acc\left(k\right)$$

For multiple NRR values, the multi-point balanced-performance efficiency (MBPE) is defined as the AUC of the SBPE.

### 7.3. Protocol for Classification Experiments

The effectiveness of the proposed DM-RC and competing methods was evaluated on the brain pattern emotional recognition task based on the SEED dataset, which includes three emotion categories: negative, neutral, and positive. After model optimization and node-retention list construction using the development subset, all evaluations were performed on the evaluation subset to determine whether VC mitigation improves classification performance and enhances the discriminability of functional networks.

Including the proposed DM-RC, six methods—observed functional network, SLF, SSD, GSLF, GLF, and DM-RC—were evaluated. A set of six NRR values, *NRR* = {1, 0.75, 0.5, 0.3, 0.2, 0.1}, was applied uniformly across all methods. Performance comparisons were conducted pairwise and analyzed across NRR values.

For each method, every recording produced a collection of VC-mitigated functional networks. From each collection, subnetworks were extracted according to different NRR values, with the resulting subnetwork representation and size determined by the corresponding method–NRR combination, as summarized in Table 2. For each combination, 30 independent classification experiments were conducted, and the final performance was reported as the average classification accuracy across these experiments.

Within each experiment, a five-fold cross-validation scheme was employed, with no information shared across different recordings or collections. No shuffle operation was performed before or after fold segmentation to prevent data leakage.

### 7.4. Deep-Learning Settings and Architecture

A minimalist CNN (Table 3) was designed for the brain pattern recognition classification experiments in this study to ensure training stability, reduce the influence of extraneous variables, and minimize the risk of introducing unintended biases. The network applies two convolutional blocks (Conv + BatchNorm + ReLU) to extract spatial features, followed by max pooling and global adaptive pooling to reduce dimensionality. The resulting 64-dimensional feature vector is flattened and passed through two fully connected layers (32 → 3 neurons). A final SoftMax layer outputs the probability for three target classes.

## 8. Model Optimization

### 8.1. Principle

To obtain an optimal VC-mitigated functional network using the proposed DM-RC, the model parameters *Θ* = {*σ*, *α*, *β*, *μ*, *k*} must be calibrated against a suitable reference.

Ideally, this reference would consist of the ground-truth functional network derived from underlying functional interactions. However, as such ground truth is inherently unobservable in scalp EEG, a task-relevant proxy—channel importance—was adopted to guide the optimization process.

This optimization does not assume that MI-based channel importance represents the ground-truth functional network. Instead, it is used as a task-relevant supervisory proxy to constrain the corrected network toward discriminative channel-level information.

For an EEG recording, channel importances were quantified using mutual information (MI) between experimental task labels and the raw EEG signal from the corresponding channel. Specifically, the MI-based task-relevant channel importance is defined as follows:

(36) $${CI}_{i}=MI\left(Task\;Labels,x_{i}\right),{CI}_{i}\in R.$$

(37) $$\mathrm{or}\;CI=MI\left(Task\;Labels,x\right),CI\in R^{C}.$$

where *i* ∈ {1, 2, …, *C*} indexes the EEG channels, and *CI_i_* refers to the importance of the *i*-th channel. *x_i_* represents the signal of the *i*-th channel within the EEG data, which was previously defined in (23).

In addition, for a functional network which was previously defined in (28) as ${FN}^{\left(e,b,n\right)}\in R^{C\times C}$, node importance is commonly adopted as a proxy for channel importance and is quantified by the average node strength. The importance of the *i*-th node is defined as follows:

(38) $${NI}_{i}=\frac{1}{C}\sum_{j=1}^{C}{FN}_{i,j},{NI}_{i}\in R,$$

(39) $$\mathrm{or}\;NI={mean}_{col}\left(FN\right),NI\in R^{C},$$

where *FN_i,j_* denotes the functional connectivity value measured between channels *i* and *j*. Accordingly, *NI_i_* refers to the importance of the *i*-th node for the functional network (i.e., the *i*-th channel of the EEG recording), and is comparable to the channel importance of (21). *mean_col_*(⋅) refers to a column-wise mean operation. Equation (39) is equivalent to Equation (30).

For the VC-mitigated functional network obtained by the DM-RC, which is defined in (15) as $FN^{\prime }(\Theta )=M\left(FN,DM;\Theta \right),$ the same formulation was applied as follows:

(40) $${NI}^{{FN}^{\prime }(\Theta )}={mean}_{col}\left({FN}^{\prime }(\Theta )\right),{NI}^{{FN}^{\prime }(\Theta )}\in R^{C}.$$

Consequently, the optimal parameters *Θ* of the modeling of DM-RC were determined by minimizing the discrepancy between the channel importance (37) and the VC-mitigated node importance (40). Formally, the optimal parameters were obtained as follows:

(41) $$\Theta^{*}=\underset{\Theta }{\mathrm{arg min}}L\left(CI,NI^{{FN}^{\prime }(\Theta )}\right).$$

where *Θ* = {*σ*, *α*, *β*, *μ*, *k*} denotes the model parameters of the DM-RC, and L(⋅) is the loss function, implemented as the mean squared error (MSE) in this study.

It should be noted that the optimization procedure was performed on recording-level average functional networks rather than on individual windows or cognitive-state-specific functional networks. Consequently, the mitigation/correction procedure applies a same optimized VC-CM to all estimated functional networks, rather than adaptively estimating a separate VC-CM for each window. The purpose of this averaging was to obtain a stable estimate of the dominant spatial pattern of VC contamination within recordings and to establish a practical engineering framework for this study. Although EEG functional connectivity exhibits temporal non-stationarity, the proposed optimization targets the recording-level characteristics of VC, which are primarily determined by relatively stable factors such as electrode geometry, inter-channel distance, and signal mixing properties.

In addition, the proposed optimization framework relies on MI-based channel importance as a task-relevant supervisory proxy rather than a direct representation of the underlying functional network. The underlying assumption is that channels carrying greater task-relevant information are more likely to occupy important positions within the true functional network, and, therefore, a higher correspondence between channel importance and network-derived node importance may indicate a more biologically meaningful network representation. However, this assumption may not always hold, since mutual information reflects the discriminative relevance to the experimental task rather than the actual strength of functional interactions. Consequently, the optimization objective should be interpreted as promoting consistency between the task relevance and network topology, rather than recovering a ground-truth functional network. Future studies may investigate alternative supervisory signals, such as source-level activity estimates, neurophysiological priors, or multimodal measurements, to further improve the biological interpretability of the optimization process.

### 8.2. Implementation

As described in Section 6.6, the EEG dataset was divided into a development subset and an evaluation subset. All computations in this section were conducted exclusively on the development subset. Although the DM-RC model represents VC components solely as a function of the inter-electrode distance, the distinct characteristics of amplitude-based and phase-based functional connectivity measures, namely, PCC and PLV, necessitated separate parameter optimization procedures. Consequently, two independent parameter sets were obtained, one optimized for PCC and the other for PLV.

For quick reference, the correspondence between principles and implementations for parameter optimization is summarized in Table 4.

First, the development subset was used to estimate the MI-based task relevant channel importance. For each EEG recording, the channel-wise MI was computed once as (37), and the final channel importance vector was obtained by averaging across recordings:

(42) $$\langle CI\rangle =\frac{1}{E_{dev}}\sum_{e=1}^{E_{dev}}MI(Task\;Labels,x^{\left(e\right)}),\langle CI\rangle \in R^{C},$$

where ⟨⋅⟩ denotes the averaging operation, $\langle CI\rangle \in R^{C}$ is a vector referring to the average channel importances, and *e* indexes EEG recordings in the development subset.

Second, to enhance robustness and stabilize the optimization process, an average for the observed functional network (PCC measurement) was constructed prior to the calculation of node importance. Specifically, functional networks were first estimated within each segment and frequency band for every recording, and then averaged across all segments, bands, and recordings:

(43) $$\langle FN\rangle =\frac{1}{EBT}\sum_{e=1}^{E_{dev}}\sum_{b=1}^{B}\sum_{n=1}^{N}{FN}^{\left(e,b,n\right)},$$

where ⟨⋅⟩ denotes the averaging operation, and $\langle FN\rangle \in R^{C\times C}$ refers to the average observed functional network.

The average functional network after VC mitigation $\langle {FN}^{\prime }\rangle \in R^{C\times C}$ was then obtained by applying the DM-RC (18) to the average observed functional network:

(44) $$\langle {FN}^{\prime }\rangle \left(\Theta \right)=M\left(\langle FN\rangle ,DM;\Theta \right).$$

The node importance derived from the average functional network ⟨*FN’*⟩ after VC mitigation was defined as the average strength and computed as the column-wise mean, the same as in (31), as follows:

(45) $${NI}^{\langle {FN}^{\prime }\rangle (\Theta )}={mean}_{col}\left({\langle FN\rangle }^{\prime }(\Theta )\right),{NI}^{{\langle FN\rangle }^{\prime }(\Theta )}\in R^{C}.$$

Finally, the optimal parameters Θ* were determined by the optimization process defined in (41), which involves (42) and (45), and were then applied as follows:

(46) $$\Theta^{*}=\underset{\Theta }{\mathrm{arg min}}L\left(\langle CI\rangle ,{NI}^{{\langle FN\rangle }^{\prime }(\Theta )}\right).$$

### 8.3. Optimal Parameters

The optimization was conducted within a bounded search space for each parameter in DM-RC construction. These bounds constrain the correction model to remain physiologically plausible and numerically stable while avoiding degenerate solutions.

The bounded search space and optimal parameters *Θ* = {*σ*, *α*, *β*, *μ*, *k*} for DM-RC are summarized in Table 5. For the parameter set *Θ_g_*, the optimal value of *k* for all models converged to the predefined upper bound of 1, indicating the reasonableness of residual correction. In the parameter set *Θ_f_*, the *σ*, *α*, and *μ* were successfully optimized, whereas *β* reached its boundary. We retained this constraint because *β* controls the steepness of the function, and extremely large or small values would cause the function to deviate from its classical shape.

**Figure 3:**
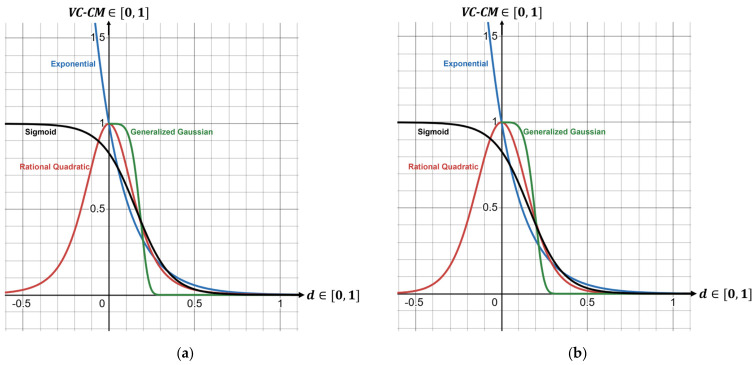
Optimized decay functions for all variants. Panel (**a**) presents the functions with parameters optimized based on PCC measurements, while panel (**b**) presents those optimized based on PLV measurements. Despite differences in the optimal parameter values, the resulting decay profiles exhibit highly consistent behaviors across the two connectivity measures. The only notable discrepancy is observed in the generalized Gaussian variant, where the PCC-based optimization yields a sharper threshold transition than the PLV-based optimization.

The decay functions with optimal parameters are shown on Figure 3. Under the unified formulation of (14), all distances and connectivity strengths were normalized to [0, 1], allowing a direct comparison of the decay behaviors across models.

The results derived from the PCC and PLV measurements were highly consistent. Despite the fact that the optimization procedures were formulated under different assumptions and applied to different types of functional connectivity measures, the estimated VC behavior and its corresponding components (i.e., the optimal parameters) showed remarkable agreement. This observation suggests that the underlying VC effects are predominantly determined by inter-electrode distances and are therefore largely independent of the specific connectivity metric used for optimization.

All decay functions exhibited their maximum attenuation at short distances (*d* = 0), with the sigmoid variant showing a slightly lower saturation level than the others. For all variants, the effective attenuation was primarily confined to the short- and mid-distance range, with decay values approaching zero beyond *d* ≈ 0.6–0.8.

Except for the generalized Gaussian, the optimized decay curves showed highly similar shapes across different functional forms, particularly for *d* > 0.2. In this region, the decay behaviors converged, suggesting a consistent attenuation pattern recovered by the optimization process. Furthermore, all variants exhibited inflection points clustered around *d* ≈ 0.2, with comparable attenuation magnitudes, indicating a common transition region between strong and weak distance-dependent VC estimation.

#### Search Bounds

- Scaling factor *k* ∈ [−1, 1]: This controls the magnitude and direction of the residual correction while preventing numerical instability or overcorrection. The optimized value *k* = −1 suggests that the VC-CM corresponds to a subtractive VC-induced component, supporting a linear decomposition of the observed functional network into functional and VC-related components.
- Decay width *σ* ∈ [0.1, 10]: This regulates the spatial decay rate in all models except the sigmoid. This range covers both localized and broadly distributed VC effects.
- Shape control parameters *α*, *β* ∈ [0.1, 5]: These control the tail heaviness or sharpness of the rational quadratic and generalized Gaussian models, balancing flexibility while avoiding overly flat or peaked decay profiles.
- Center shift *μ* ∈ [0.1, 10]: This determines the sigmoid inflection point, enabling flexible transitions while avoiding saturation caused by overly flat or steep curves.

Overall, these bounds provide sufficient model flexibility while maintaining numerical stability and computational feasibility during optimization.

### 8.4. Parameter Settings for Competing Methods

For the competing GSLF and GLF methods, the same optimization procedure was applied under an identical objective, namely, minimizing the discrepancy between the MI-based task-relevant channel importance as in (37) and the node importance derived from the average functional network after VC mitigation as in (40).

However, the VC mitigation methods used in (40) were GSLF and GLF, respectively, instead of the proposed DM-RC. The results are shown in Table 6.

#### Search Bounds

- Strength of Laplacian filtering α* ∈ [−5, 5]: In GLF, the scalar parameter α* controls the strength and direction of graph Laplacian filtering by scaling the contribution of the Laplacian operator. For the high-pass filter *F = α* * *L*, the magnitude ∣α*∣ determines the degree of high-frequency emphasis, while the sign of α controls the direction (i.e., sign inversion) of the extracted high-frequency components and spatially localized variations. The search range of [−5, 5] is chosen to symmetrically cover both positive and negative scaling regimes while avoiding numerical instability or the excessive distortion of the connectivity structure.

## 9. Evaluation Results

### 9.1. Retained Nodes/Electrodes and Subnetwork Structure

Concern arises regarding the comparative evaluation of these methods against the baselines. Specifically, comparing results across entirely separate, method-specific node-retention lists may introduce bias, thereby compromising the credibility and interpretability of the evaluation framework.

To examine the stability of node-retention lists across methods, we compared the retained electrodes derived from the development subset at *NRR* = 0.3 and *NRR* = 0.2, corresponding to 18 and 12 retained electrodes, respectively. As shown in Table 7, the DM-RC variants showed a high overlap with the observed functional networks. For PCC, the overlap ranged from 15/18 to 17/18 at *NRR* = 0.3 and from 10/12 to 11/12 at *NRR* = 0.2. For PLV, the overlap ranged from 13/18 to 16/18 at *NRR* = 0.3 and from 10/12 to 11/12 at *NRR* = 0.2. These results indicate that DM-RC did not substantially alter the high-ranking node structure, and that comparisons at identical NRR values were conducted on largely comparable subnetworks.

**Figure 4:**
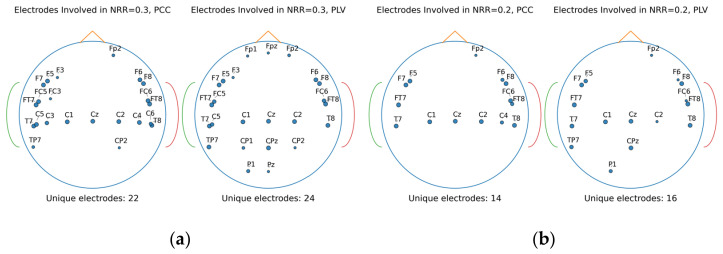
Retained nodes/electrodes derived from the observed functional network and DM-RC variants at (**a**) *NRR* = 0.3 and (**b**) *NRR* = 0.2. These maker size roughly reflects occurrence frequency for each electrode.

The corresponding electrode distributions are illustrated in Figure 4. The retained electrodes were mainly located in the frontal, fronto-central, central, and temporal regions. Several electrodes, including F7/F5/F3, F6/F8, FC5/FC6, FT7/FT8, T7/T8, and C1/Cz/C2, were repeatedly preserved across conditions, suggesting stable discriminative contributions. When *NRR* decreased from 0.3 to 0.2, the electrode set became sparser, but the overall spatial pattern remained similar. Posterior CP/P electrodes were more often retained at *NRR* = 0.3 and were mostly removed at *NRR* = 0.2, indicating less stable contributions under stronger sparsity. Compared with PCC, PLV tended to retain slightly more centro-parietal and parietal electrodes, suggesting that phase-based connectivity may provide complementary posterior information.

### 9.2. Average Accuracy of Classification Experiments

**Figure 5:**
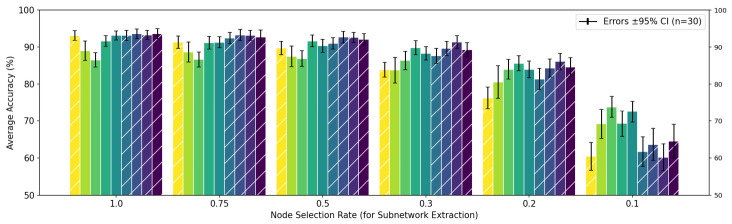
Average classification accuracy (%) on PCC under different NRRs. Error bars indicate the standard deviation. Classification accuracy decreases as the network size is reduced.

**Figure 6:**
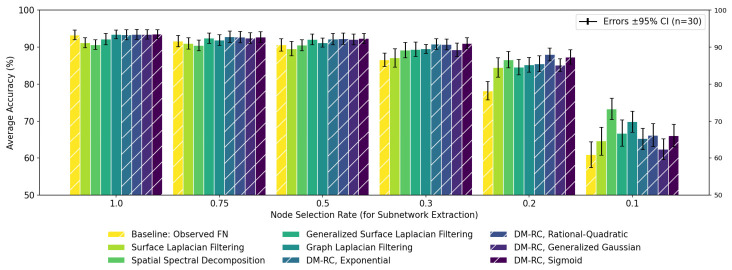
Average classification accuracy on PLV under different NRRs.

**Figure 7:**
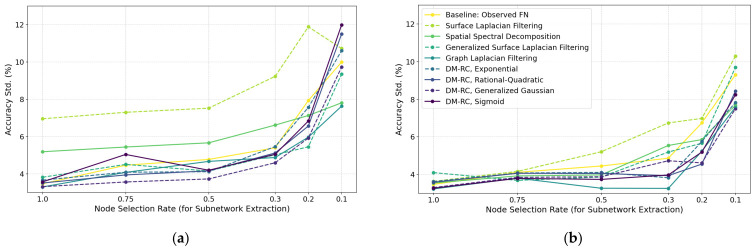
Standard deviation of classification accuracy (%) under different NRRs: (**a**) on PCC measurements; and (**b**) on PLV measurements.

**Figure 8:**
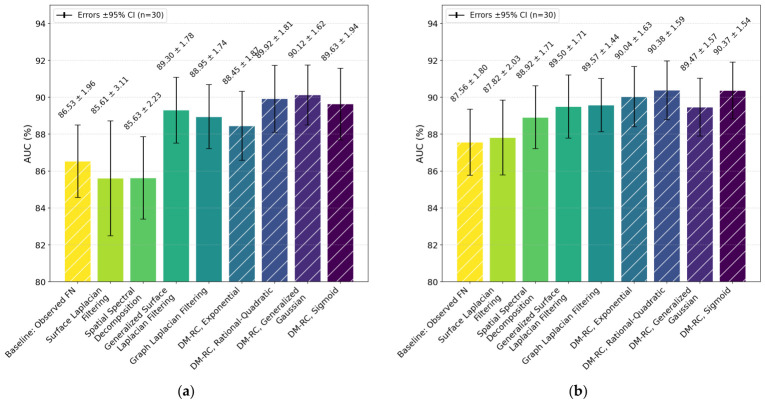
AUC of the average classification accuracy-NRR: (**a**) on PCC measurements; and (**b**) on PLV measurements. The scores follow the hierarchical ranking: (1) DM-RC, (2) network-level competitors, (3) sensor-level competitors, and (4) the baseline functional network.

For each combination of method and NRR value, 30 independent classification experiments were conducted, and the average classification accuracy is reported in Figure 5 and Figure 6, with the corresponding standard deviations shown in Figure 7a,b. To provide a more comprehensive comparison across NRR levels, the area under the curve (AUC) of the average accuracy–NRR relationship was computed for each method, as shown in Figure 8a,b.

To further evaluate the performance improvements achieved through VC mitigation, a repeated-measures ANOVA (RM-ANOVA) was performed across all classification experiments, with method and NRR treated as within-subject factors. Subsequent pairwise comparisons were conducted to further examine the observed effects.

#### 9.2.1. Results on PCC Measurements (Average Accuracy)

Classification accuracy is the most intuitive metric for evaluating the performance of different methods.

Overall, the performance of all methods degrades as the NRR value decreases, indicating the increasing difficulty of classification with smaller subnetworks. The proposed DM-RC consistently outperforms the baseline and the sensor-level competitors, while it is comparable to network-level competitors, when the *NRR* ranges from 1.0 to 0.3.

When the subnetwork size is further reduced to *NRR* = 0.2, the overall performance of DM-RC starts to degrade. Nevertheless, its variant of the generalized Gaussian still achieves top-tier performance and remains competitive with the best-performed GSLF, which yields the highest accuracy among the competing methods.

At *NRR* = 0.1, DM-RC exhibits a more pronounced performance degradation and falls below most competing methods; however, it still consistently outperforms the baseline of the observed functional network.

The standard deviation curves provide further insights into the robustness of different methods. Most methods demonstrate improved stability compared to the baseline across all NRR values. SSD generally shows weaker stability than other methods except the baseline but achieves relatively favorable performance at *NRR* = 0.2 and *NRR* = 0.1. Apart from the baseline and SSD, the differences in stability among the remaining methods are relatively minor.

#### 9.2.2. Results on PLV Measurements (Average Accuracy)

Compared with the baseline, most VC mitigation methods demonstrate higher accuracy across all NRR values. Specifically, DM-RC maintains competitive performance at higher NRR values (*NRR* > 0.1), consistently outperforming the baseline and remaining comparable to the strongest competing methods. As the subnetwork size further decreases to *NRR* = 0.1, DM-RC shows a clear performance drop and is surpassed by several competitors; however, it still consistently outperforms the baseline.

The standard deviation curves reveal additional insights. In general, most methods demonstrate a lower variability than the baseline across all NRR values, suggesting an improved robustness to subnetwork sampling. Among the competing methods, SLF exhibits relatively large performance fluctuations as NRR decreases, particularly at low NRR values, indicating a limited robustness under severe sparsification. In contrast, the DM-RC variants show more stable behavior across a wide range of NRR values, with comparatively smaller standard deviations at moderate sparsity levels (*NRR* ≥ 0.3). Although the variability of DM-RC increases at *NRR* = 0.2 and *NRR* = 0.1, its stability remains comparable to that of other methods.

#### 9.2.3. Comparison Between PCC and PLV Results

A comparison between PCC- and PLV-based results reveals several notable differences. First, the overall classification accuracy under PCC measurement is generally lower than that under PLV across all methods, indicating that PCC-based functional networks are more sensitive to node removal and subnetwork sparsification. Second, performance degradation occurs earlier and more rapidly under PCC than under PLV as the NRR decreases (at *NRR* = 0.2), highlighting the greater vulnerability of phase-based measurement to aggressive subnetwork reduction.

In terms of stability, PCC measurement tends to exhibit larger standard deviations at low NRR values compared to their PLV counterparts, suggesting an increased variability and reduced robustness. Nevertheless, DM-RC consistently maintains a favorable trade-off between accuracy and stability under both connectivity measures, outperforming the baseline and remaining competitive with advanced methods across a wide range of NRR values.

#### 9.2.4. Performance Degradation at Extremely Low NRRs

At the extreme sparsification level of *NRR* = 0.1, DM-RC exhibits a more pronounced performance degradation than several competing methods, particularly SSD and GLF. Although DM-RC still consistently outperforms the observed functional network baseline, its relative advantage becomes substantially reduced under this condition.

One possible explanation is related to the underlying modeling assumption of DM-RC. The proposed method estimates and removes VC components according to the spatial organization of the original functional network. When the NRR is relatively high, sufficient network topology is preserved, allowing the distance-dependent correction model to operate effectively. However, when only approximately 10% of nodes are retained, a large portion of the original network structure is removed, and the spatial patterns required by the model become increasingly incomplete. Consequently, the effectiveness of the distance-based correction strategy is reduced.

In contrast, SSD and GLF rely more heavily on local signal decomposition or local smoothing mechanisms, which may be less dependent on the preservation of the global network topology. As a result, these methods appear to retain better performance under extremely sparse subnetworks.

It is also important to note that *NRR* = 0.1 represents an intentionally challenging and highly constrained scenario. For the present dataset, only six channels remain available for classification at this sparsity level. Under such conditions, the functional network contains only a small fraction of the original connectivity information, and the resulting performance degradation is observed for all methods. Therefore, the *NRR* = 0.1 results should primarily be interpreted as a stress-test of methodological robustness rather than a typical application setting.

Despite this limitation, DM-RC consistently demonstrates superior or comparable performance across the broader and more practically relevant NRR range (*NRR* ≥ 0.2). These results suggest that the proposed method is particularly effective when a reasonable amount of the network structure is preserved, while future work may focus on developing more topology-preserving correction strategies for extremely sparse subnetworks.

#### 9.2.5. Generalizability Coefficient Test

Concerns regarding statistical unreliability arise when the average classification accuracy is calculated across mixed, independent subjects and independent experiments. Without an additional examination of the exact data structure (i.e., 10 subjects with 3 experiments each), this approach risks confusing the dataset with either 30 independent subjects (1 experiment each) or a single subject (30 experiments), thereby leading to pseudo replication and statistical invalidity. Here, the between-experiment variance is less of a concern, whereas the between-subject variance presents a more critical challenge. To eliminate this confusion and establish a rigorous statistical analysis, the generalizability theory (G-theory) framework was employed, and the absolute generalizability coefficient was calculated. As a metric of the dependability of measurement scores, the generalizability coefficient comprehensively accounts for both the between-subject variance and within-subject (residual) error. The results are presented in Table 8. The results obtained from the observed functional network and the rational quadratic variant of DM-RC were selected as representative samples, as the rational quadratic variant exhibits a well-balanced performance among all variants.

The results reveal that the absolute generalizability coefficients for both PCC and PLV measures remain robustly high under all conditions. This quantitative evidence proves that the statistical variance introduced by the between-subject differences has been effectively controlled and smoothed out by the multi-experiment replication design, confirming the robustness and validity of our evaluation framework.

### 9.3. Average AUC of the Accuracy-NRR

#### 9.3.1. Results on PCC Measurements (Average AUC)

As the AUC results on PCC show in Figure 8a, the network-level methods and all DM-RC variants outperform the baseline, confirming that subnetworks after VC mitigation consistently improve classification performance across varying NRR values, according to these methods.

Among the competing approaches, GLF and GSLF achieved strong overall performance, yielding average AUC values of 89.3% and 88.95%. The proposed DM-RC further improves performance, with its rational quadratic, generalized Gaussian, and sigmoid variants achieving the highest AUC scores (89.92%, 90.12%, and 89.63%). Notably, the DM-RC generalized Gaussian variant attains the best AUC on PCC, among all methods. This demonstrates that DM-RC maintains superior classification accuracy across a broad range of subnetwork sparsity levels.

This demonstrates that DM-RC maintains superior accuracy across most NRR values, despite a moderate performance drop at the extreme sparsity level of *NRR* = 0.1. The high AUC values indicate that the overall effectiveness of DM-RC is dominated by its strong performance under moderate and high NRR conditions, which are more representative of practical subnetwork extraction scenarios.

#### 9.3.2. Results on PLV Measurements (Average AUC)

Figure 8b presents the AUC results under PLV. All methods outperform the baseline, and demonstrate the effectiveness of VC mitigation.

The overall AUC values under PLV are slightly higher and more evenly distributed across methods compared with PCC. The competing methods—particularly GSLF, and GLF—achieve strong performance (89.5% and 89.57%). DM-RC again ranks among the top-tier methods. Its rational quadratic and sigmoid variants achieve the highest AUC values (90.38% and 90.37%).

Although certain competing methods, such as SSD and GLF, demonstrate stronger effectiveness at the extreme *NRR* = 0.1 level, their advantages are limited to this highly sparse setting. In contrast, DM-RC achieves a consistently strong performance across the majority of NRR values, leading to a superior integrated AUC performance.

#### 9.3.3. Relationship Between AUC and Extreme Sparsity Performance

It is worth noting that the AUC metric emphasizes the overall performance across all NRR values and is therefore less sensitive to isolated performance degradation under extreme sparsity. Although some competing methods, such as SSD and GLF, exhibit stronger performance at the extreme *NRR* = 0.1 level, their advantages are confined to this highly sparse regime. In contrast, DM-RC achieves a consistently strong performance across most NRR values, resulting in a superior integrated AUC performance.

Together, these results highlight a trade-off between the robustness to extreme node removal and VC mitigation capability. Consequently, for a given application scenario, the performance of VC mitigation methods should be evaluated at specific NRR values in accordance with engineering requirements.

Overall, the AUC analysis complements the accuracy results by showing that, while DM-RC may be less effective and robust under extreme sparsity, it delivers a more stable and superior performance across the border NRR range most relevant to practical functional network analysis.

#### 9.3.4. Behaviors of Competing Methods

A consistent performance hierarchy is observed across both measurements: network-level methods (GSLF and GLF) outperform sensor-level methods (SLF and SSD), which, in turn, outperform the baseline. However, sensor-level methods behave differently under PCC and PLV. Under PCC, they fail to surpass the baseline, indicating a limited effectiveness for correlation-based connectivity. In contrast, under PLV, signal-based methods show clear and stable improvements to the baseline, achieving higher AUC values. This suggests that sensor-level methods are better suited to phase-based connectivity than to amplitude-based correlation, whereas network-level methods remain effective and robust across both measurements.

### 9.4. RM-ANOVA and Pairwise Comparison

**Figure 9:**
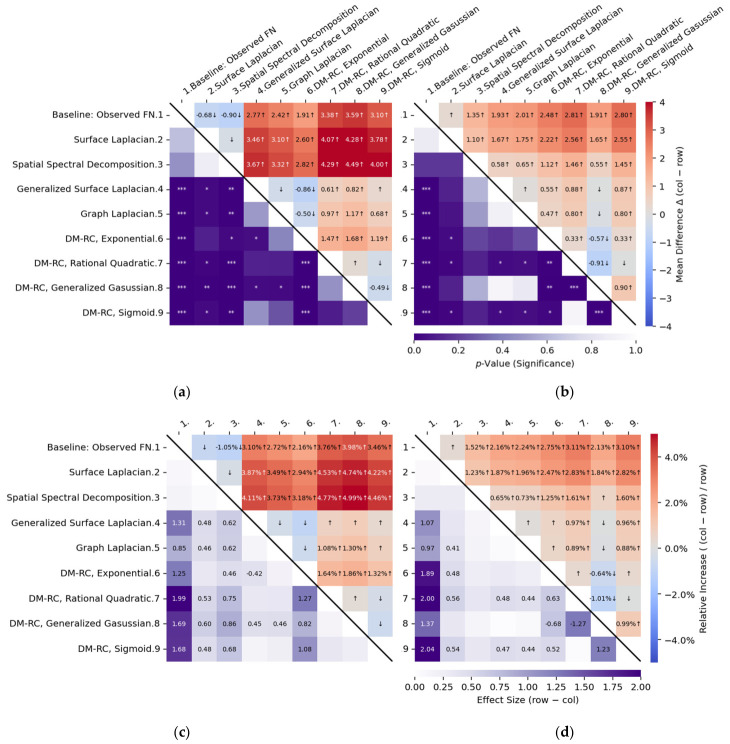
Pairwise comparison matrices for different feature extraction methods: (**a**,**b**) statistical significance maps showing the mean AUC differences (%, column minus row) and FDR-corrected *p*-values (*: *p* < 0.05, **: *p* < 0.01, ***: *p* < 0.005) from post hoc paired *t*-tests on (**a**) PCC measurements and (**b**) PLV measurements; and (**c**,**d**) performance gain maps showing the relative AUC improvements (%) and effect sizes (Cohen’s *d*) on (**c**) PCC measurements and (**d**) PLV measurements.

To further examine the performance improvements achieved by the VC mitigation methods, a RM-ANOVA was conducted. The analysis was performed across all classification experiments, with the method and NRR treated as two within-subject factors. Each was evaluated under all method–NRR combinations. The RM-ANOVA results are represented in Figure 9 and summarized in Table 9 and Table 10.

The RM-ANOVA revealed a highly significant main effect of NRR was observed (PCC: *F* = 381.13, *p* = 1.65 × 10^−81^; PLV: *F* = 568.72, *p* = 2.37 × 10^−93^), suggesting a strong influence of NRR values on the results. A significant main effect of method (PCC: *F* = 5.79, *p* = 9.75 × 10^−7^; PLV: *F* = 7.49, *p* = 6.95 × 10^−9^), indicating that the performance differed significantly across the evaluated methods, was also observed. Importantly, the method × NRR interaction was significant (PCC: *F* = 26.16, *p* = 2.81 × 10^−133^; PLV: *F* = 16.22, *p* = 1.71 × 10^−85^), demonstrating that the relative performance of the methods varied across different NRR values.

#### 9.4.1. Pairwise Comparison

Based on the confirmed significance of the method factor in the RM-ANOVA, partwise post hoc comparisons were further conducted to investigate whether specific performance improvements existed between the proposed method and the baseline/competing approaches.

The statistical and practical significance of these improvements are presented in two sets of spliced matrices. Figure 9a,b display the statistical metrics, where the mean AUC differences (Δ = column − row) are reported in the upper triangular matrix and the corresponding *p*-values are shown in the lower triangular matrix. To further evaluate the practical impact and strength of these improvements, Figure 9c,d report the relative increase percentage (*r* = (column − row)/row) in the upper triangular matrix and the corresponding Cohen’s *d* effect size in the lower triangular matrix.

Overall, the pairwise comparisons indicate that the proposed DM-RC achieves comprehensive performance improvements compared with both the baseline and competing methods across all evaluation metrics.

#### 9.4.2. Results for the Spliced Matrix Based on PCC Measurements

- Comparison against Baseline: All variants of DM-RC exhibit higher mean AUC values than the baseline (Figure 9a, row 1, columns 6–9). The corresponding *p*-values indicate highly statistically significant differences (rows 6–9, column 1; *p* < 0.005). Crucially, as shown in Figure 9c (rows 6–9, column 1), this statistical significance is supported by exceptionally large effect sizes (Cohen’s *d* ranging from +1.25 to +1.99);
- Comparison against Competing Methods: Overall, the DM-RC variants achieved higher mean AUC values than both the baseline and competing methods (Figure 9a, columns 6–9, rows 1–5), while the exponential variant lagged behind GSLF and GLF (column 6, rows 4–5). The associated *p*-values report the significance of these differences (rows 6–9, columns 1–5), and the corresponding effect sizes are reported in Figure 9c, rows 6–9, columns 1–5.

More specifically, compared with the sensor-level competing methods, SLF and SSD, the DM-RC variants show significant improvements, except for the exponential variant compared with SLF (Figure 9a, columns 6–9, rows 2–3; rows 6–9, columns 2–3). The corresponding effect sizes consistently range from positive values to as high as +0.86 (generalized Gaussian versus SSD), as reported in Figure 9c, columns 1–3, rows 6–9.

Compared with the network-level methods, GSLF and GLF, the DM-RC variants achieved higher mean AUC scores (except for the exponential variant; Figure 9a, columns 6–9, rows 4–5). However, only the generalized Gaussian variant achieved significant improvements (row 8, columns 4–5), with the corresponding medium effect sizes (0.45 and 0.46; Figure 9c, row 8, columns 4–5).

- Superiority of the Generalized Gaussian Variant: Furthermore, the generalized Gaussian variant (row 8 and column 8 in both Figure 9a,c) demonstrates a superior performance over all other approaches. According to the post hoc tests, it shows statistically significant differences compared with GSLF and GLF, which are the two best-performing competing methods (Δ = +1.17) and (Δ = +1.68), *p* < 0.05), with medium effect sizes (*d* = +0.45, *d* = +0.46). The rational quadratic and sigmoid variants also belong to the top-performing tier; however, they do not exhibit significant differences or meaningful effect sizes relative to GSLF and GLF.
- Competing Methods: Among the competing methods, the network-level methods (GSLF and GLF) consistently outperform the baseline and sensor-level methods, as reflected by positive mean AUC differences (Figure 9a, columns 4–5, rows 1–3; rows 4–5, columns 1–3) and medium-to-large effect sizes (Figure 9c, rows 4–5, columns 1–3; *d* ranging from +0.46 to +1.31).

#### 9.4.3. Results for the Spliced Matrix Based on PLV Measurements

- Overall: the comparison patterns based on PLV measurements are similar to those based on PCC; however, there are three key differences that may provide interesting insights: (1) the overall improvements achieved by DM-RC, as well as by the network-level competitors GSLF and GLF, are smaller (row 1 in both Figure 9b,d) while maintaining similar levels of significance and effect sizes (column 1); (2) the sensor-level methods, SLF and SSD, exhibit stronger effects on PLV measurements than on PCC; and, (3) although the generalized Gaussian variant performs best on PCC measurements, its performance deteriorates on PLV, whereas the rational quadratic variant, although not the best on PCC, maintains high performance and stability.
- Comparison against Baseline: All variants of DM-RC exhibit higher mean AUC values than the baseline (Figure 9b, row 1, columns 6–9). The corresponding *p*-values indicate highly statistically significant differences (rows 6–9, column 1; *p* < 0.005). Crucially, as shown in Figure 9d (rows 6–9, column 1), this statistical significance is supported by exceptionally large effect sizes (Cohen’s *d* ranging from +0.97 to +2.04).
- Comparison against Competing Methods: Overall, the DM-RC variants achieve higher mean AUC values than both the baseline and competing methods (Figure 9b, columns 6–9, rows 1–5), while the generalized Gaussian variant lags behind GSLF and GLF (column 8, rows 4–5). The associated *p*-values report the significance of these differences (rows 6–9, columns 1–5), and the corresponding effect sizes are reported in Figure 9d, rows 6–9, columns 1–5.

More specifically, compared with the sensor-level competing method SLF, the DM-RC variants show significant improvements (except for the generalized Gaussian variant; Figure 9b, row 2 and column 2). The corresponding effect sizes consistently range from positive values to a medium effect size of +0.56 (rational quadratic), as reported in Figure 9d, columns 1–3, rows 6–9. The DM-RC variants also show higher AUC scores than the sensor-level method SSD (Figure 9b, row 3); however, they do not achieve statistically meaningful significance (column 3) or effect sizes (Figure 9d, column 3).

Compared with the network-level methods, GSLF and GLF, the DM-RC variants achieved higher mean AUC scores (except for the generalized Gaussian variant; Figure 9b, columns 6–9, rows 4–5). The rational quadratic and sigmoid variants achieved significant improvements (rows 7 and 9, columns 4–5), with the corresponding medium effect sizes (ranging from +0.44 to +0.48; Figure 9d, rows 7 and 9, columns 4–5).

- Superiority of the Rational Quadratic and Sigmoid Variants: Furthermore, the rational quadratic and sigmoid variants (rows 7 and 9, columns 7 and 9, in both Figure 9b,d) demonstrate superior performance over all competing methods. They show statistically significant differences compared with GSLF and GLF, which are the two best-performing competing methods (Δ ranging from +0.80 to +0.88, *p* < 0.05), with medium effect sizes (*d* ranging from +0.41 to +0.48). The exponential variant also belongs to the top-performing tier; however, it does not exhibit significant differences or meaningful effect sizes relative to GSLF and GLF. The generalized Gaussian variant performs best on PCC measurements; however, its performance deteriorates on PLV.
- Competing Methods: Among the competing methods, the network-level methods (GSLF and GLF) consistently outperform the baseline and sensor-level methods, as reflected by positive mean AUC differences (Figure 9b, columns 4–5, rows 1–3; rows 4–5, columns 1–3) and effect sizes ranging from positive to large (Figure 9d, rows 4–5, columns 1–3; *d* up to +1.07). However, they lose their dominance over the sensor-level methods, as indicated by the lack of statistical significance and meaningful effect sizes. This may be due to the effectiveness achieved by the sensor-level methods on PLV measurements, in contrast to PCC (Figure 9b, row 1, columns 2–3).

#### 9.4.4. Performance Differences of DM-RC in PCC and PLV Measurements

The relative improvements achieved by DM-RC are smaller for PLV than for PCC, and several pairwise comparisons lose statistical significance. This finding suggests that DM-RC is more compatible with amplitude-based connectivity measures than with phase-based connectivity measures.

This is likely explained by the differences in the susceptibility of connectivity measures to VC contamination. Two observations support this interpretation: 1. The baseline performance of the observed PLV networks is already higher than that of the observed PCC networks (AUC scores, Figure 9, PLV: 87.56%, PCC: 86.53%), suggesting that PLV provides intrinsically clearer connectivity estimates than PCC. This advantage may be attributable to its greater robustness to artifacts and noise, a substantial portion of which may arise from VC. 2. Not only DM-RC but also the competing network-level methods (GSLF and GLF) achieve smaller improvements for PLV than for PCC. Therefore, the reduced gains are not unique to DM-RC; instead, they appear to reflect a general characteristic of network-level VC mitigation when applied to phase-based connectivity measures.

In contrast, the sensor-level methods (SLF and SSD) exhibit a different pattern: they improve performance when applied to PLV measurements, but their performance on PCC measurements is even lower than the baseline performance of the observed networks. These methods mitigate VC at the sensor level, while also modifying the original EEG signals and may remove information associated with genuine neural activity. Such signal distortions may affect PCC and PLV differently, leading to variations in their relative performance across connectivity measures.

Importantly, despite the smaller gains observed for PLV, both DM-RC variants still significantly outperform the observed PLV networks and remain competitive with, or superior to, existing network-level methods in several comparisons (Figure 9b,d). Therefore, the results support the conclusion that DM-RC is effective for both amplitude-based and phase-based functional connectivity measures, while providing greater benefits for amplitude-based measures, where VC contamination appears to be more pronounced.

### 9.5. Results of BPE

**Figure 10:**
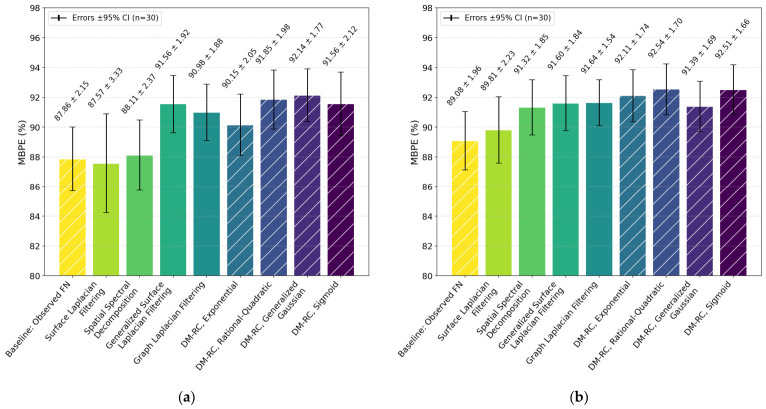
MBPE of the average classification accuracy-NRR: (**a**) on PCC measurements; and (**b**) on PLV measurements. The scores follow the hierarchical ranking: (1) DM-RC, (2) network-level competitors, (3) sensor-level competitors, and (4) the baseline functional network.

**Figure 11:**
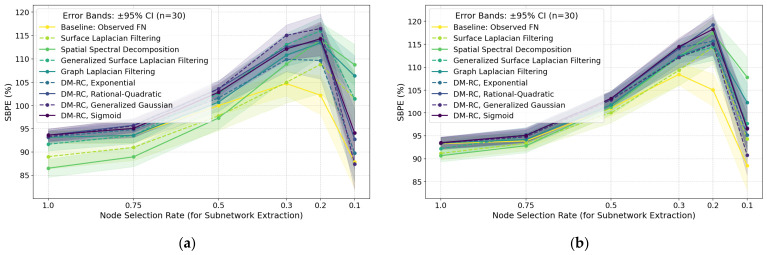
SBPE of the average classification accuracy-NRR: (**a**) on PCC measurements; and (**b**) on PLV measurements.

The results of MBPE and SBPE under different measurements of PCC and PLV are shown in Figure 10 and Figure 11.

The patterns of MBPE are similar to the AUC results: DM-RC substantially outperforms the baseline and is comparable with GLF and GSLF. An interesting pattern is observed for SSD: it is weaker than the baseline in the AUC comparison under PCC but stronger than the baseline in the MBPE comparison. This confirms the effectiveness and significance of SSD, a classical method for VC mitigation, which do not work as expected at larger network sizes but are effective at smaller sizes, which are often adopted in practical BCI applications.

The SBPE patterns of the methods are similar, and all the methods consistently outperform the baseline across NRR values. There are fewer conclusions that can be drawn into this figure; however, it is interesting to note that all the methods reach a consensus that NRR, with the best performance efficiency, is between 0.2 to 0.3; although this insight may be restricted in our definition of SBPE and the used dataset, this procedure for ascertaining the best SMPE can be considered to instruct the construction of practical BCI systems.

The SBPE patterns of the methods are similar, and all methods consistently outperform the baseline across NRR values. Fewer conclusions can be drawn from this figure; however, one interesting observation is that all methods reach a consensus that the NRR with the best performance efficiency lies between 0.2 and 0.3. Although this insight may be restricted to our definition of SBPE and the dataset used, this procedure for ascertaining the best SBPE can be considered to inform the construction of practical BCI systems.

### 9.6. Visual Insights Based on DM-RC

Beyond the overall performance, the specific spatial distributions and mitigation effects of the estimated components merit closer examination.

**Figure 12:**
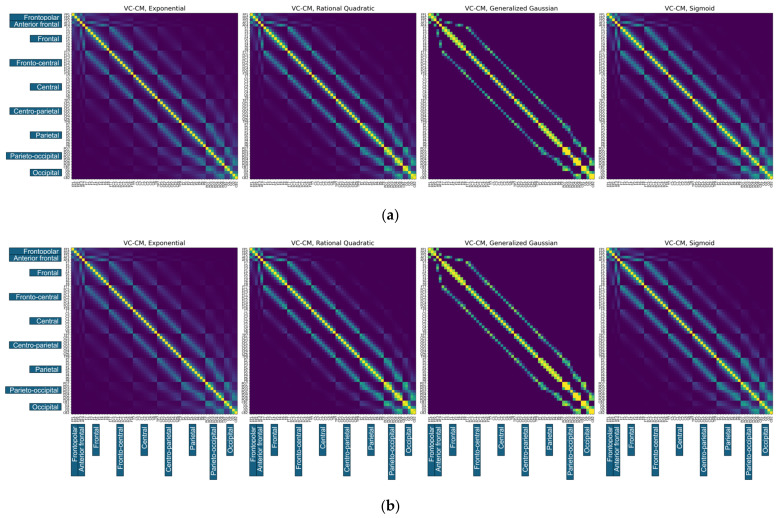
The estimated VC components (i.e., VC-CM) between EEG electrodes obtained using different variants of DM-RC. Panel (**a**) based on PLV and panel (**b**) based on PCC connectivity measures.

Figure 12 shows the estimated VC components derived from DM-RC using PLV (panel a) and PCC (panel b) functional networks. Both connectivity measures produce highly similar spatial patterns, indicating that the estimated VC structure is largely independent of the synchronization metric and primarily reflects the physical characteristics of volume conduction.

Across all variants, VC strength is concentrated near the main diagonal and decreases with inter-electrode distance, forming characteristic banded structures. Stronger VC effects are observed within anatomical regions (frontal, central, parietal, and occipital), whereas interactions between distant regions are substantially weaker, consistent with the spatially local nature of VC.

The exponential, rational quadratic, and sigmoid variants produce similar smooth distance-dependent VC distributions. In contrast, the generalized Gaussian yields a much sparser pattern, emphasizing dominant local interactions while suppressing weaker long-range contributions, suggesting a more selective representation of VC effects.

**Figure 13:**
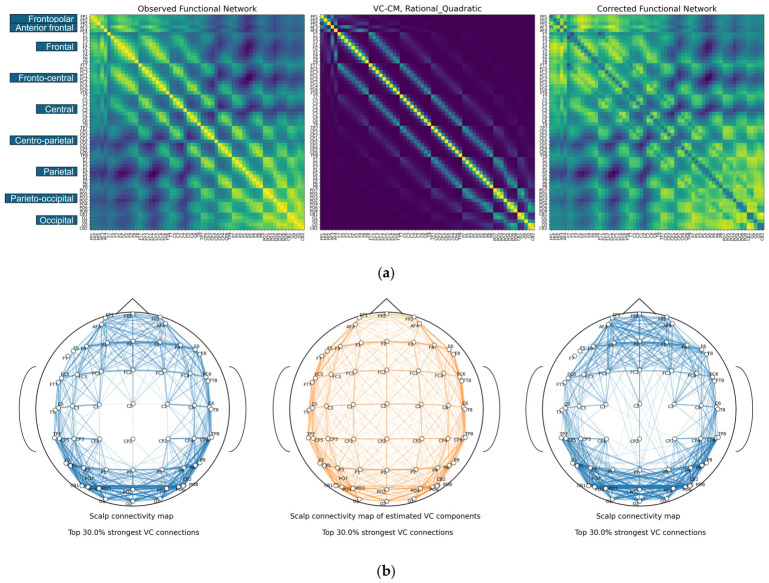
Observed functional network of the alpha band based on PCC measurements (globally averaged across the developmental subset), the estimated VC-CM using the rational quadratic kernel, and the corresponding VC-mitigated functional network. Panel (**a**) shows the functional connectivity matrix, while panel (**b**) presents the scalp connectivity map. The alpha band, PCC measurement, and rational quadratic variant are presented as a representative example.

Figure 13 illustrates the observed functional network, the estimated VC component matrix (i.e., VC-CM), and the corresponding VC-mitigated functional network using the rational quadratic kernel. The alpha band and PCC connectivity are presented as representative examples because lower-frequency activity is generally more susceptible to volume conduction effects, while the VC-CM estimates obtained from PCC and PLV exhibit highly similar spatial patterns. The rational quadratic was selected as the sample because of its balanced performance among all DM-RC variants.

In the observed network, strong connectivity is concentrated around the main diagonal and between neighboring electrode groups, producing prominent banded structures in the connectivity matrix and dense short-range connections in the scalp map. These patterns closely resemble the estimated VC-CM, suggesting that a substantial portion of the observed connectivity is associated with spatially local VC effects.

These observations can be regarded as additional evidence supporting the ability of DM-RC to mitigate VC, particularly in the sequential scalp connectivity maps shown in Figure 13b. However, they do not reveal VC-related characteristics beyond those already established in the current literature. Further insights may be obtained in future studies by extending the DM-RC framework toward window-adaptive modeling or cognitive/emotional state-specific modeling, rather than relying on the current static representation of VC components.

## 10. Discussion

The brain pattern recognition experiments evaluated the classification accuracy across methods and NRR values, the AUC of accuracy-NRR relationships, and RM-ANOVA with pairwise comparisons. Together, these analyses highlight the performance patterns of conventional VC mitigation methods and the proposed DM-RC across different connectivity measurements and model variants.

### 10.1. Major Findings (Superior Performance of DM-RC for VC Mitigation)

First, the results for the classification accuracy, AUC of accuracy–NRR, and pairwise comparisons clearly establish a hierarchical performance structure among all evaluated methods. For VC mitigation, the proposed DM-RC, GSLF, and GLF are considered network-level methods, whereas SLF and SSD are regarded as sensor-level methods. Under most conditions, DM-RC consistently outperforms the competing network-level methods (GSLF and GLF), which, in turn, achieve a superior performance compared with the sensor-level methods (SLF and SSD). All VC mitigation methods significantly outperform the baseline (the originally observed functional network).

In DM-RC, the VC components are explicitly defined and modeled using decay functions and are subsequently suppressed through a residual correction operation at the functional network level. The achieved robust and superior performance highlight the hierarchical and complementary contributions of decay modeling and network-level residual correction embodied in DM-RC, and confirm that DM-RC effectively integrates both aspects to counteract VC effects.

Second, regarding the PCC measurement, the generalized Gaussian variant of DM-RC achieves the best performance, yielding the highest AUC value and statistically significant improvements over all competing methods. This advantage stems from the flexibility of the generalized Gaussian; its shape parameter allows for the precise adaptation to the heterogeneous correlation distributions typically found in PCC-based functional networks.

However, its performance on the PLV measurement is comparatively weaker, suggesting that the distributional characteristics of phase-based connectivity differ substantially from those of amplitude-based correlations. In particular, because the parameter optimization was tailored to the PCC functional network, the model’s inherent flexibility may have inadvertently led to overfitting to that specific metric. This specialized tuning renders the model less adaptive to PLV, resulting in the observed performance degradation.

Third, although the generalized Gaussian achieves the highest AUC value for PCC, pairwise statistical analysis indicates that the rational quadratic and sigmoid variants offer a superior trade-off between performance and stability across different connectivity measurements. These two variants consistently outperform competing methods while maintaining robustness across both PCC and PLV measurements. In contrast, the exponential yields the weakest results; this suggests that constrained models—which are more inflexible in their behavior—may be insufficient to capture the complex, inherent patterns of VC components.

### 10.2. Performance Degradation of DM-RC at Extreme Sparsity Subnetwork Size

At the extremely sparsity level of *NRR* = 0.1, DM-RC exhibits a more pronounced performance degradation compared with some competing methods, particularly SSD and GLF. This behavior can be attributed to the fundamental design of DM-RC, which emphasizes global structure consistency rather than robustness to extreme sparsity.

Specifically, DM-RC relies on sufficient node support to reliably model global connectivity patterns. By suppressing globally shared components as (17), its global aggregation mechanism tends to produce representations with reduced structural contrast. Under extremely low node retention (*NRR* = 0.1), the remaining network structure becomes insufficient to sustain discriminative global patterns, leading to an effectively smooth and less informative representation. In contrast, SSD and GLF place greater emphasis on spectral or local structural variations. In particular, GLF is a graph high-pass filtering operation, which preserves coarse-grained discriminative features even under severe node removal, resulting in a comparatively better performance at *NRR* = 0.1.

However, it is important to note that the superior performance of SSD and GLF at this extreme sparsity level comes at the cost of reduced adaptability and representational richness at moderate and higher NRR values. Across the majority of NRR settings (*NRR* ≥ 0.2), DM-RC and GSLF consistently outperform these methods, indicating that it is better suited for scenarios where a reasonable level of the network structure is preserved.

Overall, the results at *NRR* = 0.1 highlight a trade-off between extreme sparsity robustness and VC mitigation capability. While SSD and GLF demonstrate a stronger tolerance to severe node removal, DM-RC provides a more effective and stable performance across practical sparsity levels, which are more relevant in practical functional network analysis and BCI applications.

### 10.3. Performance Differences of VC Methods on Different Connectivity Measurements

A consistent performance improvement is observed when moving from PCC to PLV across all evaluated methods. This trend is reflected not only in higher classification accuracy and AUC values, but also in improved stability as indicated by the reduced standard deviation. These results suggest that PLV provides a more effective and robust representation to VC components for functional connectivity, benefiting a wide range of modeling strategies. This is a consistent result revealed by former studies [8,21].

### 10.4. Spatial Characteristics of VC Decay Revealed by Optimized Functions

The decay functions with optimal parameters are shown in Figure 3, allowing a direct comparison of the decay behaviors across models.

Except for the sigmoid variant, all decay functions are aligned at *f*(0) = 1 (VC estimation), indicating a shared assumption that the strongest VC estimation occurs at zero inter-electrode distance.

The sigmoid curve exhibits a slightly lower initial value (≈0.85), reflecting its more conservative and smooth behavior, which is consistent with its overall milder and adaptive correction effect. In contrast, the generalized Gaussian employs a more aggressive strategy, consistently assigning the highest weight to the VC among all variants before the inflection point, with a near-hard cutoff later. While this radical behavior likely accounts for the generalized Gaussian’s superior performance in PCC measurements, it also introduces fragility; this trade-off is one of the probable causes for its unexpected degradation in PLV.

Across all variants, the effective decay region is concentrated within *d* ∈ [0, 0.6] or *d* ∈ [0, 0.8], suggesting that the optimization process implicitly identifies VC components beyond this range as negligible. This observation provides a data-driven estimate of the spatial support of VC effects under normalized distance.

Notably, except for the generalized Gaussian, the remaining decay functions exhibit highly similar profiles, indicating that the optimization converges toward an eye-catching identical behavior. This convergence enhances the credibility of the learned decay behavior and suggests a shared understanding of VC components and mitigation strategies across different functional forms.

A more detailed inspection reveals that, while noticeable differences exist in the very short-range regime (*d* < 0.2), the decay curves rapidly converge for *d* > 0.2. This pattern implies that, although different variants adopt distinct strategies for handling near-neighbor interactions, a consensus emerges in modeling VC components beyond a certain critical distance.

Furthermore, all variants exhibit inflection points around *d* ≈ 0.2, with the corresponding VC estimation falling within a narrow range of approximately [0.3, 0.5]. This consistency suggests the existence of a potential transition region where VC effects undergo a qualitative change, providing a promising reference for future function design or piecewise modeling strategies.

### 10.5. Limitations of Sensor-Level Methods

#### 10.5.1. Limitation of SLF

Although SLF is designed to suppress VC-driven spatial leakage by subtracting a weighted combination of neighboring signals, its formulation inherently introduces a risk of signal cancellation. This phenomenon has been documented in former studies [33,34].

Specifically, when removing the leakage from neighboring signals in channel *i*, the neighboring signals V(k),k∈N(i) contain not only the signal potential from channel *k* but also components originating from channel *i* itself.

In such cases, the subtraction term $\sum_{k\in N(i)}W_{i,k}V(k)$ suppresses *V(k)*, while removing the *V(i)* components that are embedded in *V(k)*, leading to the suppression of the desired *V(i)* potential.

Moreover, even when the weights *W_i,k_* are distance-informed and locally constrained, the operation still constitutes a form of wide-range subtraction. This can induce an implicit global shrinkage of co-fluctuation amplitudes across signals, compressing the dynamic range of inter-signal dependencies and, in turn, weakening the structural separability of the resulting representations (functional connectivity).

Importantly, these effects can be exacerbated when the distances, neighborhood definition, or weight estimation are biased or mismatched to the actual spatial leakage, which increases the probability that SLF removes not only the leakage-dominated components but also the desired signal potential.

#### 10.5.2. Limitation of SSD

A similar limitation can also be observed for SSD. Although SSD is formulated as a frequency-domain method that maximizes the target-band variance relative to flanking bands via a generalized eigenvalue problem, the “noise” covariance *Cn* constructed from neighboring frequency bands is not guaranteed to be independent of the target-band activity. In realistic EEG recordings, neural sources are often broadband and subject to spectral leakage; consequently, the flanking-band signals used to form *Cn* may still carry projections of the same underlying sources that dominate the target band. This perspective aligns with previous observations in [31].

This violates the idealized separation between *Cs* and *Cn*, and can again induce the attenuation of genuine target-band information when SSD suppresses directions that also explain the variance in the flanking bands. In addition, the SSD reconstruction *X’ = (W^†^)^⊤^ W^⊤^ X* can be interpreted as applying a linear projection operator to the raw signals, which enhances a subset of components while compressing or discarding others. As a result, SSD may also introduce an implicit shrinkage of the covariance structure—particularly for amplitude-based dependencies—thereby reducing the discriminative contrast of resulting representations (functional connectivity) derived from the transformed signals. A similar concern has been raised previously [35]. Like SLF, SSD is sensitive to the bias in model choices (e.g., flanking band placement, the number of bands *K*, and regularization strength), and suboptimal settings may amplify both signal attenuation and structural compression.

#### 10.5.3. The Common Limitation

Although SLF and SSD operate in different domains—the sensor-level spatial domain and the frequency domain, respectively—their suppression of neighboring components of the desired signal shares a common risk: the neighboring components may themselves contain contributions from the desired signal due to spatial mixing or broadband source characteristics. This shared concern has been highlighted across several studies [31,33,34,35]. As a result, the subsequent subtraction or contrast optimization may suppress not only leakage-dominant components but also components from the desired signal when they are embedded in the neighborhood, leading to the degradation of the desired signal itself.

This process can induce an implicit global shrinkage of co-fluctuation amplitudes across signals, thereby compressing the dynamic range of inter-signal dependencies. Such an effect is particularly pronounced for PCC, which directly relies on the amplitude co-variation, compared with PLV, which is less sensitive to amplitude attenuation. This difference may explain why SLF and SSD can improve PLV-based connectivity while yielding weaker or less discriminative functional networks when applied to PCC.

These observations support the view that improvements achieved by sensor-level suppression or enhancement methods are not guaranteed to propagate to downstream feature engineering stages, especially in functional connectivity estimation, where the impact of preprocessing can be strongly feature-dependent [19].

In contrast to sensor-level methods, network-level methods do not attempt to eliminate VC components at the signal source; instead, they operate directly on functional matrices. This design is more compatible with functional-network-based BCI pipelines in terms of both the workflow and computational efficiency, while also offering improved interpretability, comparability, reproducibility, and scalability.

## 11. Methodological and Framework Contributions

### 11.1. Methodological Contributions

#### 11.1.1. Functional-Network-Level Modeling

VC-induced contamination is fundamentally a network-level phenomenon. Its most salient consequences include inflated zero-lag correlations, elevated global node strength, and reduced contrast between genuine functional interactions and spurious connections. These effects are not directly apparent in raw EEG signals, but become explicit once interactions are summarized as functional networks.

Operating directly on functional connectivity therefore targets the final manifestation of VC rather than its presumed upstream causes. Whereas signal-level spatial filtering attempts to attenuate smooth spatial components before the connectivity is computed, functional-network-level approaches operate on the distorted connectivity itself, where VC is already measurable and analyzable. This makes the functional-network level a more natural domain for characterizing, modeling, and mitigating VC-induced contamination.

This choice is also well-aligned with downstream EEG analysis. In many applications, including classification, subnetwork analysis, and graph-based learning, functional networks are the actual inputs to predictive models. When VC mitigation is performed only at the signal or source level, its effects on connectivity remain indirect and difficult to control. By contrast, a direct correction at the functional-network level makes it possible to evaluate how mitigation influences classification accuracy, subnetwork robustness, and graph-theoretic properties such as node strength, sparsification behavior, and performance-efficiency trade-offs.

A further advantage of functional-network-level operation is that it is non-destructive with respect to the original signals. Unlike signal- or source-level processing, which irrevocably alters the underlying time series, functional-network-level transformations act only on derived representations while leaving the original EEG intact. This separation provides additional flexibility in both analysis and system design.

Because the original signals are preserved, VC mitigation at the functional-network level need not be treated as a purely subtractive operation. Instead, the enhanced functional network can be used together with the observed one to construct enriched representations of the form $F(FN,{FN}^{\prime })$, where the enhanced network captures structures revealed by inverse modeling, while the observed network may still retain informative global or low-frequency interaction patterns.

From this perspective, VC mitigation is not only a denoising problem but also a representation-enrichment problem. Functional-network-level mitigation naturally supports multi-view representations, whereas signal-level filtering irreversibly removes components that may still contain task-relevant information. In this sense, functional-network-level approaches are not only suitable for VC mitigation, but also advantageous for scalable and flexible EEG representation learning.

#### 11.1.2. Stability and Efficiency Gained Through Temporal Integration

Functional networks possess a defining property that distinguishes them from signal- and source-level representations: they compress temporal information into a compact statistical object. Connectivity measures such as correlation and phase synchrony aggregate interactions over extended time windows, and, in some cases, across trials, yielding a single matrix representation. This temporal integration reduces sensitivity to instantaneous fluctuations, sensor noise, and trial-level variability.

By contrast, signal- and source-level VC mitigation operates directly on time-resolved data. Filtering or inverse procedures must therefore be applied across large numbers of samples, making them more sensitive to transient perturbations and nonstationarity. Even minor signal-level noise may propagate through spatial filters or inverse operators, leading to unstable results across trials or recording sessions [36,37].

Functional networks, in contrast, represent temporally aggregated interaction patterns, allowing corrective operations to act on more statistically stable structures. This can reduce the variance in the corrected representation and improve robustness, particularly when operating on sparse or reduced subnetworks.

Temporal integration also provides a computational advantage. Signal- and source-level processing typically scales with both the number of channels *C* and the number of time samples *T*, resulting in complexity on the order of *O(C × T)* or higher when filtering or inverse operations are repeatedly applied over time. At the functional-network level, the temporal dimension is collapsed before correction, so VC mitigation is performed directly on matrices of size *C × C*. Once the functional network has been constructed, subsequent operations depend primarily on *C*, yielding complexity on the order of *O*(*C*^2^), independent of *T*.

This shift from time-dependent to structure-dependent computation is especially advantageous in EEG and BCI pipelines, where long recordings and high sampling rates can otherwise dominate the processing cost. By decoupling VC mitigation from the temporal dimension, functional-network-level approaches offer a greater statistical stability and a lower computational cost while preserving the network structure used in downstream analysis.

#### 11.1.3. Information Efficiency Under Weak Geometric Assumptions

A central design choice in this study is the adoption of a weak geometric assumption, whereby only electrode-level spatial information (e.g., inter-electrode distances) is used, without the reliance on subject-specific head models or detailed forward solutions. Under this constraint, functional connectivity represents an information-efficient abstraction layer.

Source-level methods require substantial additional priors that may not be reliably available in practical settings. Functional network level operation preserves interaction information while requiring only modest geometric input. Each piece of spatial information contributes directly to modeling or correcting connectivity distortions, rather than serving as an indirect or approximate proxy for unobserved sources.

This efficiency is particularly advantageous in real-world BCI and affective computing scenarios, where individualized anatomical data are unavailable and computational resources are limited.

### 11.2. Evaluation Framework Contributions

#### 11.2.1. Classification-Based Framework for Method Evaluation

To evaluate the method’s effectiveness, this study incorporates an objective and robust evaluation pipeline. This pipeline uses actual neural-pattern classification experiments and integrates 30 independent classification runs, 95% confidence intervals, paired statistical tests, relative-increase measures, and effect-size analyses. This comprehensive framework provides strong empirical support and operates under a key assumption: if functional network recovery translates into improved discriminability for downstream neural decoding, then, conversely, the improved classification performance demonstrates the recovery of genuine functional networks. Importantly, this approach avoids the common pitfall of relying purely on subjective evaluations, such as visual or qualitative assessments of connectivity. Furthermore, this rigorous classification-based framework is generalizable and can be applied to evaluate other artifact mitigation methods (e.g., for EMG or EOG artifacts) or enhancement methods, thereby reducing subjective bias in the performance assessment.

#### 11.2.2. Subnetwork Extraction and Balanced-Performance-Efficiency Evaluation

A novel methodological contribution is the introduction of the subnetwork-extraction × BPE framework. By jointly considering node retention rates, classification accuracy, and computational savings, this hybrid evaluation captures the trade-offs essential for real-time BCI systems. The discovery of an optimal performance-efficiency point near 20% NRR suggests that full-sized functional networks may be over-parameterized and that moderate sparsification enhances both robustness and efficiency. This observation aligns with recent findings that sparse or pruned graphs often generalize better in high-dimensional neuroimaging data.

This framework has the potential to become a general tool for assessing connectivity-based methods in practical BCI development.

## 12. Conclusions

This study proposed a functional-network-level volume conduction (VC) mitigation method, termed decay modeling–residual correction (DM-RC), which explicitly models spatially induced VC-related components using inter-electrode distance information and suppresses them through residual correction. Unlike conventional sensor-level or source-level methods, DM-RC operates directly on functional connectivity representations (functional matrices), enabling post hoc correction without reprocessing raw EEG signals and reducing computational overhead.

By introducing task-relevant channel importance as a weak supervisory reference, DM-RC aligns network-level node importance with cognitive relevance, allowing a principled optimization of model parameters under realistic constraints. Experimental results on the SEED dataset demonstrate that DM-RC consistently improves classification performance in brain pattern recognition tasks across a wide range of subnetwork sizes and connectivity measurements, outperforming both sensor-level and network-level competing methods in overall accuracy, robustness, and area-under-the-curve (AUC) metrics.

Although performance degradation is observed under extreme sparsity conditions, the results indicate that DM-RC provides a favorable trade-off between VC mitigation effectiveness and network structural stability in practical functional network analysis scenarios. Overall, the proposed DM-RC offers an efficient, interpretable, and engineering-friendly solution for VC mitigation, and holds promise for practical functional-connectivity-based pattern recognition BCIs.

At the same time, the introduction of the distance matrix, the functional-network-level perspective, and the corresponding operational framework, and the novel evaluation framework based on classification experiments, subnetwork extraction, and balanced-performance efficiency provide important theoretical and methodological implications, offering new directions for neuroscience research and future BCI applications.

## Figures and Tables

**Table 1:** Employed decay functions and corresponding expressions.

Models	Expression	Parameters
Exponential	$f\left(d\right)=e^{-(\frac{d}{\sigma })}$	*σ*
Rational Quadratic	$f\left(d\right)={\left(1+\frac{d^{2}}{{2\alpha \sigma }^{2}}\right)}^{-\alpha }$	*σ*, *α*
Generalized Gaussian	$f\left(d\right)=e^{-({\frac{d}{\sigma })}^{\beta }}$	*σ*, *β*
Sigmoid	$f\left(d\right)=\frac{1}{1+e^{\frac{d-\mu }{\beta }}}$	*β*, *μ*

**Table 2:** Data structure from EEG signals to functional-network feature representations used in the deep-learning classification framework.

Level	Full Dataset	Development Subset	Evaluation Subset
EEG	$EEG\in R^{E{\times (Lf}_{s})\times C}$	/	/
Segmented EEG	$EEG\in R^{E\times (NT)\times C}$ 45 × (3394 × 200) × 62	${EEG}_{dev}\in R^{E_{dev}\times (NT)\times C}$ 15 × (3394 × 200) × 62	${EEG}_{eva}\in R^{E_{eva}\times (NT)\times C}$ 30 × (3394 × 200) × 62
Functional networks	$FN\in R^{E\times N\times B\times \left(C\times C\right)}$ 45 × 3394 × 3 × 62 × 62	${FN}_{dev}\in R^{E_{dev}\times N\times B\times C\times C}$ 15 × 3394 × 3 × 62 × 62	${FN}_{eva}\in R^{E_{eva}\times N\times B\times C\times C}$ 30 × 3394 × 3 × 62 × 62
Model optimization	/	Ditto	/
Node retention list estimation for subnetwork extraction	/	Ditto	/
Deep-learning framework for classification experiments	/	/	Collections: ${FN}_{eva}\in R^{E_{eva}\times (N\times B\times C\times C)}$ 30 × 3394 × 3 × 62 × 62 *E_eva_* experiments: ${FN}^{\left(e\right)}\in R^{N\times (B\times C\times C)}$ 3394 × 3× 62 × 62 *N* sample: ${FN}^{\left(e,n\right)}\in R^{B\times C\times C}$ 3 × 62 × 62
Deep-learning framework for classification experiments (on subnetworks)	/	/	Collections: Ditto *E_eva_* experiments:$\;\;{FN}^{\left(e\right)}\in R^{N\times (B\times NRR\cdot C\times NRR\cdot C)}$ 3394 × 3 × *NRR* 62 × *NRR* 62 $N\;\mathrm{sample}:\;{FN}^{\left(e,n\right)}\in R^{B\times (NRR\cdot C\times NRR\cdot C)}$ 3 × *NRR* 62 × *NRR* 62

**Table 3:** Deep-learning architecture.

Stage	Operation	Details
Input	Input + Per-channel Instance Normalization	Collection of functional networks *N × B × C × C* (3394 × 3 × 62 × 62) Note: Normalization is performed independently within each 62 × 62 feature map.
Conv Layer 1	Conv + BN + ReLU	3 × 3, 32 filters, stride = 1, padding = 1
Pooling 1	Max Pooling	3 × 3, stride = 3
Conv Layer 2	Conv + BN + ReLU	3 × 3, 64 filters, stride = 1, padding = 1
Pooling 2	Adaptive Max Pooling	Output size = 1 × 1 (Global pooling)
Flatten	Flatten	Convert to 1D vector (64)
FC Layer 1	Fully Connected	32 neurons
FC Layer 2	Fully Connected	3 neurons
Output	SoftMax	3-category classification

**Table 4:** Directory of formulations for parameter optimization.

Level	Channel Importance (Reference)	Node Importance (Model)	Optimization
Principle	CI=MI(Task Labels,x)	$NI={mean}_{col}\left(FN\right)$	Θ* arg minΘLCI,NIFN′(Θ)
Implementation	$\langle CI\rangle =\frac{1}{E_{dev}}\sum_{e=1}^{E_{dev}}MI(Task\;Labels,x^{\left(e\right)})$	${NI}^{{\langle FN\rangle }^{\prime }(\Theta )}={mean}_{col}\left({\langle FN\rangle }^{\prime }(\Theta )\right)$	Θ* = arg minΘLCI,NIFN′(Θ)

**Table 5:** Parameters and search bounds for optimal model.

Feature Domain	Model	*Θ_g_*	*Θ_f_* for Decay Function			
		*k* ∈ [−1, 1]	*σ* ∈ [0.1, 10]	*α* ∈ [0.1, 5]	*β* ∈ [0.1, 5]	*μ* ∈ [0.1, 10]
PCC	Exponential	1	0.173712			
	Rational Quadratic	0.99918	0.129823	2.6162		
	Generalized Gaussian	1	0.193288		4.9957	
	Sigmoid	0.9953			0.1006	0.15829
PLV	Exponential	1	0.171083			
	Rational Quadratic	0.99991	0.145752	5.743876		
	Generalized Gaussian	0.99978	0.205384		4.996665	
	Sigmoid	0.99903			0.100372	0.159782

**Table 6:** Parameters and search bounds for optimal model.

Feature Domain	Model	Expression	*σ* ∈ [0.1, 10]	α* ∈ [−5, 5]
PCC	GSLF	$W_{\left(i,j),(k,l\right)}=\exp\left(-\frac{\left(DM_{i,k}+DM_{j,l}{)}^{2}\right.}{2\sigma^{2}}\right)$	2.020393	
	GLF	$F=\alpha^{*}L$	0.242139	−1.43394
PLV	GSLF		1.728712	
	GLF		0.25134	−4.65221

α* is the parameter used in Laplacian filtering and is introduced to distinguish it from the previously defined ***α*** in the proposed DM-RC method.

**Table 7:** Node/electrode retention lists derived from the observed functional network and DM-RC variants at sample NRRs.

*NRR* = 0.3 (Number of Channels: 18)										*NRR* = 0.2 (Number of Channels: 12)									
PCC					PLV					PCC					PLV				
Obs.	Exp.	RQ	GG	Sig.	Obs.	Exp.	RQ	GG	Sig.	Obs.	Exp.	RQ	GG	Sig.	Obs.	Exp.	RQ	GG	Sig.
					Fp1														
					Fpz														
Fp2			Fp2		Fp2			Fp2		Fp2					Fp2				
F7	F7	F7	F7	F7	F7	F7	F7	F7	F7	F7	F7	F7	F7	F7	F7	F7	F7	F7	F7
F5	F5	F5	F5	F5	F5	F5	F5	F5	F5	F5	F5	F5	F5	F5	F5		F5	F5	F5
			F3					F3											
F6	F6	F6	F6	F6	F6	F6	F6	F6	F6			F6	F6	F6				F6	
F8	F8	F8	F8	F8	F8	F8	F8	F8	F8	F8	F8	F8	F8	F8	F8	F8	F8	F8	F8
FT7	FT7	FT7	FT7	FT7	FT7	FT7	FT7	FT7	FT7	FT7	FT7	FT7	FT7	FT7	FT7	FT7	FT7	FT7	FT7
FC5	FC5	FC5	FC5	FC5	FC5		FC5	FC5	FC5										
		FC3																	
FC6	FC6	FC6	FC6	FC6	FC6		FC6	FC6	FC6	FC6	FC6	FC6	FC6	FC6				FC6	
FT8	FT8	FT8	FT8	FT8	FT8	FT8	FT8	FT8	FT8	FT8	FT8		FT8		FT8	FT8	FT8	FT8	FT8
T7	T7	T7	T7	T7	T7	T7	T7	T7	T7	T7	T7	T7	T7	T7	T7	T7	T7	T7	T7
C5	C5	C5	C5	C5		C5	C5	C5	C5										
C3	C3	C3		C3															
C1	C1	C1	C1	C1	C1	C1	C1	C1	C1	C1	C1	C1	C1	C1	C1	C1	C1	C1	C1
Cz	Cz	Cz	Cz	Cz	Cz	Cz	Cz	Cz	Cz	Cz	Cz	Cz	Cz	Cz	Cz	Cz	Cz	Cz	Cz
C2	C2	C2	C2	C2	C2	C2	C2	C2	C2	C2	C2	C2	C2	C2		C2			
C4	C4	C4	C4	C4							C4	C4		C4					
	C6		C6	C6															
T8	T8	T8	T8	T8	T8	T8	T8	T8	T8	T8	T8	T8	T8	T8	T8	T8	T8	T8	T8
TP7	TP7				TP7	TP7	TP7	TP7	TP7						TP7	TP7	TP7		TP7
							CP1		CP1										
					CPz	CPz	CPz	CPz	CPz						CPz	CPz	CPz	CPz	CPz
				CP2		CP2													
						P1	P1		P1							P1	P1		P1
						Pz													
18/18	17/18	16/18	15/18	16/18	18/18	13/18	15/18	16/18	15/18	12/12	11/12	10/12	11/12	10/12	12/12	10/12	11/12	10/12	11/12
Overlap ratio to the list derived from original PCC										Overlap ratio to the list derived from original PLV									

Obs.: observed functional network; Exp.: exponential variant of DM-RC; RQ: rational quadratic; GG: generalized Gaussian; Sig.: sigmoid.

**Table 8:** Absolute generalizability coefficient across NRRs, for the observed functional network and the rational quadratic variant of DM-RC.

Measure	Method	NRR					
		1	0.75	0.5	0.3	0.2	0.1
PCC	Observed	0.893457	0.860872	0.875279	0.87892	0.883701	0.897309
	Rational quadratic	0.874048	0.882158	0.87602	0.88622	0.889636	0.896066
PLV	Observed	0.883755	0.885756	0.890029	0.879272	0.881076	0.896622
	Rational quadratic	0.87138	0.889098	0.894952	0.874827	0.891808	0.887864

**Table 9:** Repeated ANOVA of different methods across NRRs, on PCC.

	F Value	Pr > F	Num DF	Den DF
Method	5.79	9.75 × 10^−7^	8	232
NRR	381.13	1.65 × 10^−81^	5	145
Method × NRR	26.16	2.81 × 10^−133^	40	1160

**Table 10:** Repeated ANOVA of different methods across NRRs, on PLV.

	F Value	Pr > F	Num DF	Den DF
Method	7.49	6.95 × 10^−9^	8	232
NRR	568.72	2.37 × 10^−93^	5	145
Method × NRR	16.22	1.71 × 10^−85^	40	1160

## Data Availability

The original contributions related to parameter optimization and classification performance evaluation presented in this study are included in the article. Further inquiries can be directed to the corresponding author. Restrictions apply to the availability of the EEG dataset used in this study. The data were obtained from the SEED team and are available from the SEED project [38] with permission from the dataset developers.
